# Origin of Correlations between Local Conformational States of Consecutive Amino Acid Residues and Their Role in Shaping Protein Structures and in Allostery

**DOI:** 10.1021/acs.jpcb.2c04610

**Published:** 2022-11-11

**Authors:** Celina Sikorska, Adam Liwo

**Affiliations:** † The MacDiarmid Institute for Advanced Materials and Nanotechnology, Department of Physics, The University of Auckland, Private Bag 92019, Auckland1142, New Zealand; ‡ Faculty of Chemistry, University of Gdańsk, Fahrenheit Union of Universities in Gdańsk, Wita Stwosza 63, 80-308Gdańsk, Poland

## Abstract

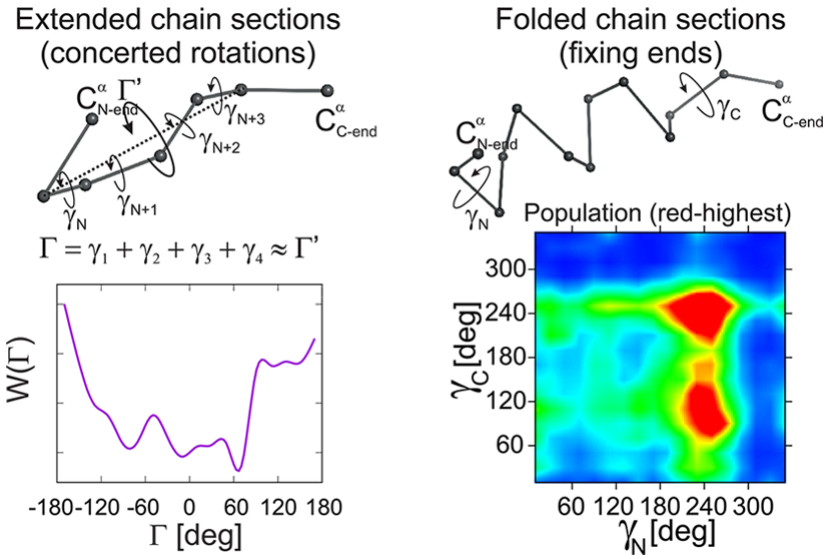

By analyzing the Kubo-cluster-cumulant expansion of the potential of mean force of polypeptide chains corresponding to backbone-local interactions averaged over the rotation of the peptide groups about the C^α^···C^α^ virtual bonds, we identified two important kinds of “along-chain” correlations that pertain to extended chain segments bordered by turns (usually the β-strands) and to the folded spring-like segments (usually α-helices), respectively, and are expressed as multitorsional potentials. These terms affect the positioning of structural elements with respect to each other and, consequently, contribute to determining their packing. Additionally, for extended chain segments, the correlation terms contribute to propagating the conformational change at one end to the other end, which is characteristic of allosteric interactions. We confirmed both findings by statistical analysis of the virtual-bond geometry of 77 950 proteins. Augmenting coarse-grained and, possibly, all-atom force fields with these correlation terms could improve their capacity to model protein structure and dynamics.

## Introduction

Even though the physical principles that govern the self-organization of protein molecules into globular structures are known, physics-based protein-structure prediction is way behind the knowledge-based methods^1−3^ of which Deep Mind’s AlphaFold^4^ and AlphaFold2^5,6^ have recently made a quantum jump in the field, demonstrating outstanding performance in the 14th Community Wide Experiment on the Critical Assessment of Techniques for Protein Structure Prediction (CASP14).^5−7^ The reason for the poor performance of the physics-based methods, compared with the knowledge-based ones, especially AlphaFold2, is the inaccuracy of force fields and the large size of the systems studied. First-principles modeling of protein structure (based on the Schrödinger equation or Density Functional Theory) is beyond reach. The data- and bioinformatics-unassisted modeling with all-atom force fields, though reaching the experimental resolution for some proteins, is restricted to small proteins (about 100 amino acid residues), even when using high-performance computers, including those which have been built especially to run molecular simulations.^8−10^ Coarse graining enables us to extend the modeling scale by orders of magnitude;^11−13^ however, it is at the further expense of accuracy.

Force-field inaccuracy is certainly a more severe problem in the coarse-grained approach, compared to the all-atom modeling. In the coarse-grained approaches, most of the degrees of freedom are considered implicitly and the atomistically detailed interactions need to be absorbed in the effective potentials. However, even the all-atom force fields are only analytical approximations to the Born–Oppenheimer energy surfaces. These approximations are a trade-off between accuracy and computational efficiency (e.g., the multibody terms are usually omitted). This observation suggests that it is still not understood how elementary interactions are translated into the formation and stabilization of biomolecular structures. The physics-based coarse grained approaches, in which a closer look on how the atomistic-detailed interactions collectively form the effective interaction potentials is necessary, can contribute to this understanding.^12,14^ A good example is the theory of helix–coil transition developed by Poland and Scheraga,^15^ which is based on a very simple coarse-grained model with sequential interactions. This theory has made a big contribution to the understanding of the formation and stability of helical segments of proteins, both at the qualitative level and at a semiquantitative level.

In our recent work, we developed a scale-consistent theory of the derivation of coarse-grained force fields,^12,16^ which originates from our earlier developed Kubo cluster-cumulant expansion^17^ of the potential of mean force of a system under study, in which the degrees of freedom not explicitly included in the model (including the solvent degrees of freedom if a CG model implies implicit solvent) are averaged out.^18^ In our approach, the degrees of freedom to average over are implicitly present in the effective energy functions, accounting for nonspherical symmetry of the effective interaction potentials and for the multibody terms and also enabling us to derive the respective analytical formulas. We applied the approach to the UNRES coarse-grained models of polypeptide chains,^19−22^ which we have been developing for a long time together with the late Professor Harold A. Scheraga, obtaining better results than with the previous variants of UNRES.^23,24^ However, for larger proteins, knowledge-based restraints are needed for UNRES to be predictive.^24^

Modeling accuracy drops with the size of a system. In our recent work,^24^ we compared the performance of modeling with unassisted UNRES, UNRES assisted by predicted contacts or fragments derived from bioinformatics-based models, and AlphaFold2. In all three cases, the modeling accuracy, quantified as the Global Distance Test Total Score (GDT_TS),^25^ decreases with the number of amino acids residues exponentially, the exponent being the greatest (0.52) for unassisted UNRES and the smallest for AlphaFold2 (0.11). The decrease of accuracy with protein size suggests that the errors inherent in the method accumulate but also that AlphaFold2 and other knowledge-based methods can compensate for the errors, most probably by inferring the correlations between the geometric features of remote segments of protein structures. These long-range correlations can be the missing terms in the force fields, which are essential for correct modeling, especially with the coarse-grained approaches. Even in the coarse-grained force fields that include explicit coupling terms, such as CABS^26^ and UNRES,^19−22^ the correlations extend only to short chain segments.

The scale-consistent formalism developed in our earlier work^12,16^ enables us to find the long-range coupling terms. In this work, we employ this formalism in finding the “along-chain” terms, which correspond to the coupling between local conformational states of consecutive residues, which are manifested as multitorsional-like potentials. These terms account for the “through-virtual-bond” interactions, similar to the “through-bond” interactions introduced by Surján et al.^27^ to explain the origin of the torsional potentials at the all-atom level.

In our earlier work,^16^ we derived the scale-consistent formulas for the double-torsional potentials. In this work, we extend this derivation to the multiple-torsional potentials, which also depend on the respective virtual-bond-angles. We demonstrate that, for extended segments bordered by turns (usually β-strands), the dominant terms depend on the sum of all virtual-bond-dihedral angles of a segment, which is close in value to the dihedral angle formed by the backbone-virtual bond preceding the strand, the axis of the segment, and the virtual bond succeeding the segment. For folded spring-like (usually α-helical) segments, the multitorsional potentials depend on the products of the trigonometric functions of the virtual-bond-dihedral angle preceding a segment, those along a segment, and that succeeding a segment. In both cases, these coupling terms direct the chain before and after a segment; however, the virtual-bond-dihedral angles will change along the chain in a concerted manner only for extended chain segments. We confirm these theoretical predictions by an analysis of protein structures. Based on the results, we propose multitorsional terms that describe the long-range sequential correlations for use in coarse-grained force fields to improve their capacity to model protein structure and dynamics. We also discuss the role of the sequential correlations in determining the packing of secondary-structure elements and in indirect allosteric interactions.

## Methods

### Scale-Consistent Formulas for Multiple Torsional Potentials

**Figure 1:**
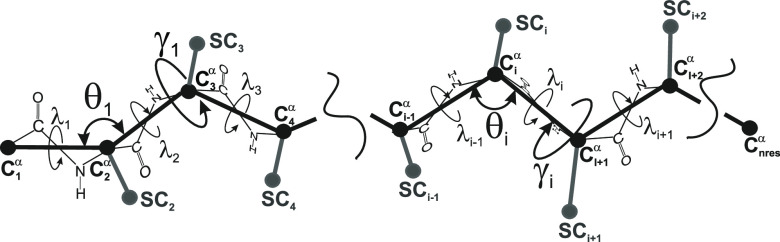
Scheme of a coarse-grained polypeptide chain that consists of *nres* residues, including the N- and C-terminal blocking groups. The backbone is defined as the α-carbon (C^α^) trace. United side chains are attached to the C^α^ atoms. All peptide groups are assumed in the *trans* configuration, this implying the C^α^···C^α^ virtual-bond length of about 3.8 Å. The backbone geometry is thus defined in terms of the virtual-bond angles θ_2_, θ_3_, ..., θ_*nres*–1_ (each carrying the index of the respective C^α^ at its vertex) and virtual-bond dihedral angles γ_2_, γ_3_, and γ_*nres*–2_ (each carrying the index of the first C^α^ atom at its axis). The potential of mean force (PMF) of the chain is obtained by integrating out the Boltzmann factors in the angles λ_1_, λ_2_, ..., λ_*nres*–1_ for the rotation of the peptide groups (shown as small atom symbols and thin lines) about the respective C^α^···C^α^ virtual-bond axes.

**Figure 2:**
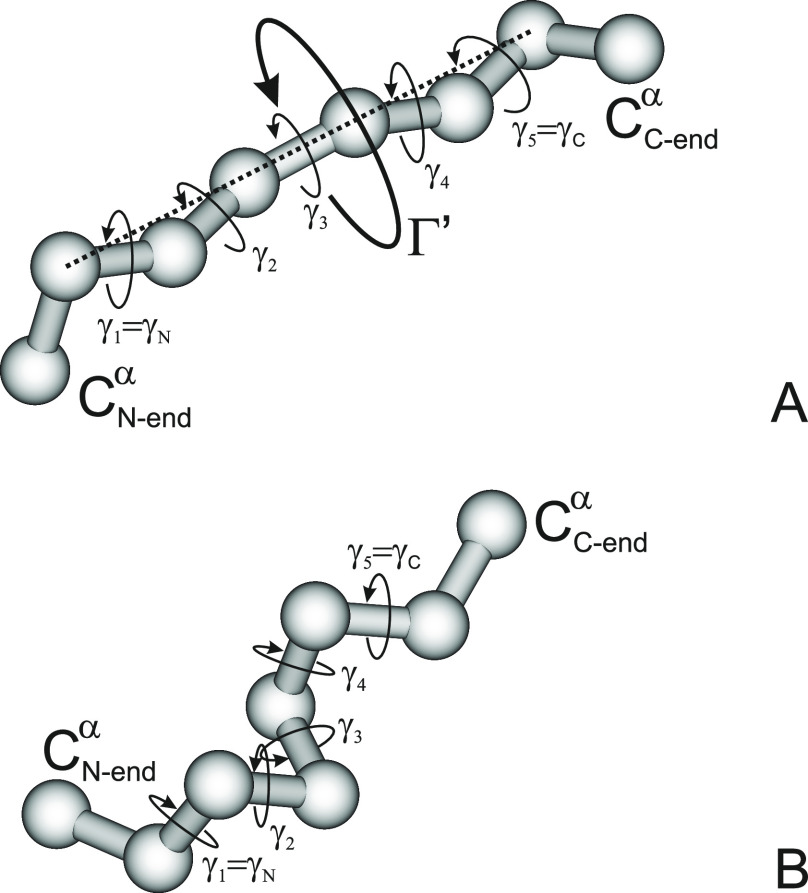
Illustration of (A) the extended turn bordered (ETB) and (B) folded (FD) chain segments with a segment of polypeptide chains comprising 8 consecutive C^α^ atoms, which are represented as gray spheres, while the C^α^···C^α^ virtual bonds are represented as gray cylinders. The five virtual-bond-dihedral angles (γ_1_, γ_2_, ..., γ_5_) are indicated. In panel A, the virtual-bond angles except those at the N-end (γ_*N*_) and at the C-end (γ_*C*_) of the chain are large, while the angles at the ends are small. The dashed line runs along the extended-part axis. The dihedral angle Γ′, defined by the terminal virtual bond and the strand axis is approximately equal to the sum of all five dihedrals along the segment. In panel B, the virtual-bond angles, except those at the ends of the segment, are small.

**Figure 3:**
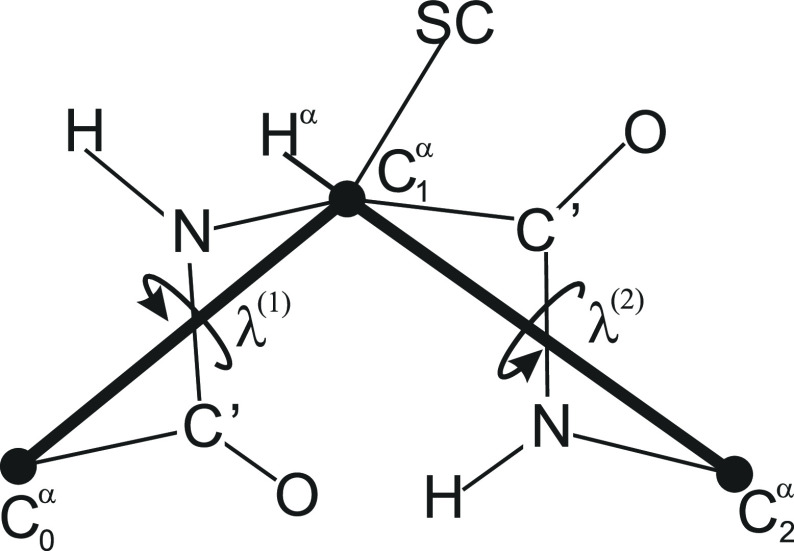
Definition of the λ^(1)^ and λ^(2)^ angles for the rotation of the peptide groups about the C^α^···C^α^ virtual-bond axes.^28^ λ^(1)^ is the angle for counterclockwise rotation of the peptide group located between C_0_^α^ and C_1_^α^. λ^(1)^ = 0 when the carbonyl-carbon atom of the peptide group is in the C_0_^α^···C_1_^α^···C_2_^α^ plane and faces C_2_^α^. λ^(2)^ is the angle for counterclockwise rotation of the peptide group located between C_1_^α^ and C_2_^α^. λ^(2)^ = 0 when the amide-nitrogen atom of the peptide group is in the C_0_^α^···C_1_^α^···C_2_^α^ plane and faces C_0_^α^. Adapted with permission from ref (18). Copyright 2001 AIP Publishing.

As in UNRES,^19−22^ we represent the polypeptide backbone, containing *nres* – 2 full residues, by its α-carbon (C^α^) trace (from C_1_^α^ to C_*nres*_^α^) with united side chains attached to the C^α^-atoms by virtual bonds (Figure 1). Here we consider only the local interactions, that is interactions within an amino acid residue, including its side chain. To simplify the considerations, we will assume that the only the interactions with the β-carbons of the side chains except for glycine, which does not have a side chain, and proline, for which the interactions with the proline-ring atoms are included; this assumption follows the philosophy of the UNRES model, in which only three residue types—glycine, proline, and other—are defined for the purpose of local interactions.^16,20^ Thus, the respective potential of mean force, *W*, is obtained by integrating the Boltzmann factor over the angles λ_1_, λ_2_, ..., λ_*nres*–1_ for the rotation about the C^α^···C^α^ virtual-bond angles, as given by eq 1. Assuming that all peptide groups are in the *trans* configuration, *W* is a function of the backbone virtual-bond angles θ_2_, θ_3_, ..., θ_*nres*–1_ and the backbone-virtual-bond-dihedral angles γ_2_, γ_3_, ..., γ_*nres*–2_ (see Figure 2 for illustration of the systems considered here).
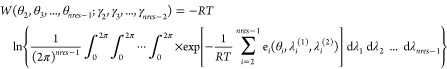
1where *e*_*i*_ denotes the local-interaction energy surface of the *i*th residue, which depends on the virtual-bond-angle θ_*i*_ and the angles λ_*i*_^(1)^ and λ_*i*_^(2)^ for the rotation about the first and the second virtual bond of the *i*th residue^28^ (Figure 3), *R* is the universal gas constant, and *T* is the absolute temperature. For a polypeptide chain with *trans*-only peptide groups, the angles λ_*i*_^(1)^ and λ_*i*_^(2)^ are related to the angles λ_*i*_ and λ_*i*+1_ by eq 2 (see ref (28)).
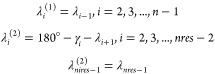
2

By generalizing the formula of the double-torsional potentials given by eq 90 of ref (16) (in which we replaced the sine and cosine terms with phase-shifted-cosine terms), we can express the generic (*m* – 2)-unit correlation part of the multitorsional potentials encompassing the segment of the chain from C_*k*_^α^ to C_*k* + *m*_^α^, *m* > 2, by eq 3. The derivation of this equation is presented in the section *Derivation of the lowest-order term in multitorsional potentials* of the Supporting Information.
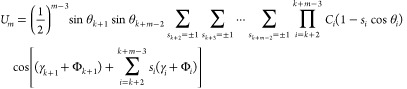
3where the phase angles Φ_*k*+1_,Φ_*k*+2_, ..., Φ_*k*+*m*–3_ depend on the all-atom geometry of the polymer units; for a polypeptide chain, primarily on residue chirality. For symmetric units, all Φs are 0 or 180° (ref (16)). The coefficients *C*_*i*,*m*_*i*__, *i* = 1, 2, ..., *k* + *m* – 2, depend on the derivatives of the energy of interactions between the respective atoms in the interatomic distance (cf eq 43 of ref (16)).

**Figure 4:**
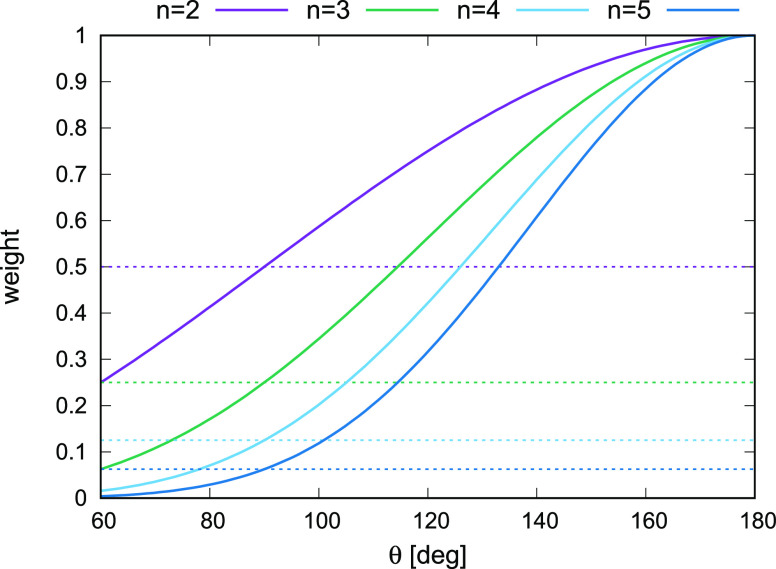
Plot of the dominant weight of the cosine terms in eq 3 for chain segments with length from 5 (*n* = 2 dihedrals) to 8 (*n* = 5 dihedrals), for which the first and the last virtual-bond angles θ are equal to 90° and the angles inside have the same value (θ) in θ. The horizontal lines correspond to the inner θ equal to 90°, in which case the weights of all multitorsional terms are equal.

Thus, the multitorsional potentials are weighted sums of the cosines of the linear combinations of the phase-shifted backbone-virtual-bond-dihedral angles γ, the coefficients being ±1 and the weights depending on the backbone-virtual-bond angles θ. The sin θ terms at the ends tend to 0 as the chain-segment ends become linear, thus preventing the torsionals from being undefined.^16^ The (1 – cos θ) terms tend to 2 and not to 0 as the chain segment (except for the ends) becomes linear. It can clearly be seen that the contribution to the sum in eq 3 with all (1 – cos θ) terms will dominate in the weighted sum for θ_*k*+2_ through θ_*k*+*m*–3_ equal nearly 180° (Figure 4); then the terms (1 – cos θ) equal 2, thus giving 1 when multiplied by the 2^–(*m*–3)^ factor in front of the sum. However, θ_*k*+1_ and θ_*k*+*m*–2_ must be away from 180° or the *U*_*m*_ will vanish. This situation is illustrated in Figure 2A and corresponds, e.g., to an β-strand bordered by turns. The *U*_*m*_ can then be approximated by eq 4.
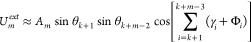
4
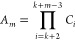
5

On the other hand, when θ_*k*+2_,θ_*k*+3_,···,θ_*k*+*m*–3_ are close to 90° (Figure 2B), a situation encountered in α-helices, the weights of all terms in eq 3 are approximately equal and *U*_*m*_ is defined by eq 6.
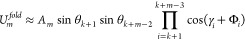
6which can be obtained from eq 3 by recursively applying the reduction formula cos(*x* + *y*) + cos(*x* – *y*) = 2 cos(*x*) cos(*y*), starting from the end of the terms sharing the same signs at angles in the cosine terms but the last one.

The sum of the angles in the multitorsional potential of turn-bordered extended chains in eq 4 is approximately equal to the torsional angle Γ′ between the flanking virtual bonds and the axis of the extended segment of the chain (Figure 2A). This means that there is a mean-field ordering term that determines the twist of the chain segments preceding and following the strand. Collective angular variables in polypeptide-backbone segments were also detected, based on the dark-soliton concept, by Niemi and co-workers.^29,30^ Moreover, the two γ angles of a 3-residue turn-bordered extended chain should be anticorrelated. For the folded (helical) segment of the chain, the multitorsional potential will direct the chain segments preceding and succeeding the helix, but in an uncorrelated manner. In the Results and Discussion, we demonstrate how the derived multitorsional potentials are reflected in protein structures.

### Protein Structure Analysis

We analyzed the virtual-bond angles θ and the virtual-bond-dihedral angles γ (Figure 1) computed from a total of 77,950 protein structures containing 157,922 chains from the Protein Data Bank (PDB; https://www.rcsb.org/, as of August 23, 2022).^31^ The structures have been selected to satisfy the following criteria: (i) resolution is explicitly defined, (ii) the resolution is 2.0 Å or better, and (iii) the structure is not an NMR structure, since NMR restraints could be absent or scarce in the loop regions which could, in turn, result in a significant contribution from the force field and conformational-search procedure applied to process the NMR data to part of the respective structures. The list of all PDB entries including chain IDs is in File S1 of the Supporting Information. The angles θ and γ were calculated from the C^α^ coordinates. The segments with *cis* amino acid residues have been omitted. One- and two-dimensional histograms of the angles were constructed with the bin in θ, *Δθ* = 10° and the bin in γ, *Δγ* = 20°.

An (*m* – 2)-residue segment of the chain containing *n* = *m* – 3 consecutive virtual-bond-dihedral angles γ, which encompasses *m* consecutive C^α^ atoms from C_*k*_^α^ to C_*k*+*m*–1_^α^, is defined as *extended turn-bordered* (ETB) if θ_*k*+1_ < 100°, θ_*k*+*m*–2_ < 100° and θ_*i*_ > θ_*ext*_, *i* = *k* + 2,*k* + 3, ..., *k* + *m*–3. We used two cutoff values of θ_*ext*_, equal to 120° and 135°, respectively. Conversely, a segment of the chain is defined as *nonextended turn-bordered* (NETB), if θ_*k*+1_ < 100°, θ_*k*+*m*–2_ < 100°, θ_*i*_ ≤ 120°, *i* = *k* + 2, *k* + 3, ..., *k* + *m* – 3, and residues from *k* + 1 through *k* + *m* – 2 are not in the HELIX records. The latter condition was introduced to eliminate the possible bias due to helices, which dominate folded segments of protein chains.

For the ETB and NETB chain segments, we constructed the 2D distributions of the terminal γ_*k*+1_ and γ_*k*+*m*–3_ virtual-bond-dihedral angles, hereafter referred to as γ_*N*_ and γ_*C*_, respectively (eq 7) and the distributions (eq 9) and the respective dimensionless potentials of mean force in the sums of the virtual-bond-dihedral angles γ(Γ) along the chain segments, normalized to be contained within [−180°, 180°](eq 11).
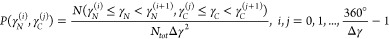
7
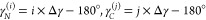
8
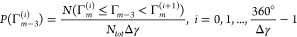
9
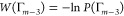
10
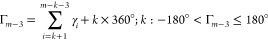
11where *i* and *j* are bin indices, the *N*s are the respective numbers of counts in a one- or a two-dimensional bin, respectively, and *N*_*tot*_ is the total number of counts. Angle superscripts in parentheses denote bin indices, as opposed to the indices of the angles in the chain, which are in subscripts. The total number of counts corresponding to all types of chain segments are summarized in Table 1.

**Table 1:** Counts of Extended Turn-Bordered (ETB), Non-Extended Turn-Bordered (NETB), Folded (FD), Folded Helical (FH), and Non-Structured (NS) Chain Segments in the Database of 77 950 Protein Structures Used in This Work for Different Segment Lengths

	*n*^a^	all	Gly and Pro excluded
ETB	2	429935	288515
	3	128357	70537
	4	38964	16887
	5	17418	6528
	6	9747	3991
	7	6413	2147
NETB	2	705453	412477
	3	210901	88759
	4	59611	20843
	5	16898	4800
	6	6488	1409
	7	3397	471
FD	2	802402	472462
	[3, 20]	1535674	606681
	>20	73014	11455
FH	2	604039	358960
	[3, 20]	1040668	283307
	>20	143356	11182
NS	2	353793	154961
	[3, 20]	1302822	270411
	>20	23322	146

a The number of dihedral angles in the segment, which is equal to *m* – 3, where *m* is the number of C^α^ atoms.

For better illustration of the relationship between the γ angles, we calculated and plotted the respective covariances, which are defined by eq 12
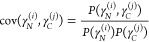
12
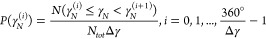
13
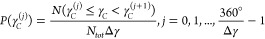
14

An (*m* – 2) -residue segment of the chain, that encompasses *m* consecutive C^α^ atoms from C_*k*_^α^ to C_*k*+*m*–1_^α^, is defined folded (FD) if θ_*i*_ < 100°, *i* = *k* + 2, *k* + 3, ..., *k* + *m* – 3, no restrictions being imposed on θ_*k*+1_ or θ_*k*+*m*–2_. Additionally, we define a chain segment as *folded helical* (FH) if θ_*i*_ < 100°, *i* = *k* + 2, *k* + 3, ..., *k* + *m* – 3, and γ_*k*+2_ through γ_*n*–3_ are contained within the interval from 0° to 70° or that the residues with indices from *k* + 1 through *k* + *m* – 2 are in the HELIX records of a respective PDB entry.

For the folded chain segments, we constructed the 2D distributions of the terminal γ angles (eq 7) and those of the terminal θ angles and the adjacent terminal γ angles (eq 15). For reference, we carried out the same analysis for the segments of chains in which all residues were not in HELIX or SHEET records; these segments are termed the *nonstructured* (NS) chain segments.
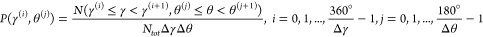
15

The above definitions of the four kinds of chain segments (ETB, NETB, FD, and FH) are unique given the θ_*cut*_ value in the definition of ETB chain segments. In particular, if a segment is shifted in amino acid sequence, it fails to satisfy the respective definition.

## Results and Discussion

### Extended Turn-Bordered Chain Segments

**Figure 5:**
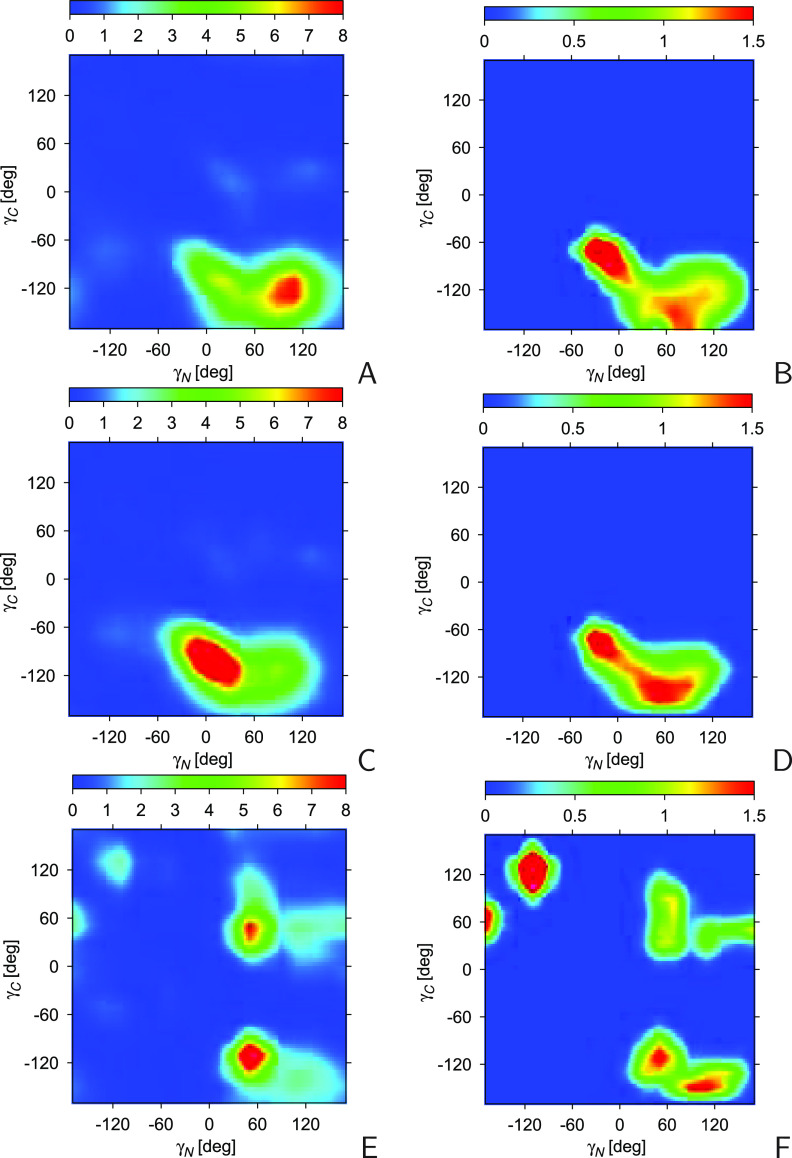
Heat maps of the 2D-distributions (A, C, and E) and correlations (B, D, and F) of γ_*N*_ and γ_*C*_ for 5-residue ETB segments of proteins chains derived assuming the cutoff for the central θ angle, θ_*cut*_ = 120° (A and B) and θ_*cut*_ = 135° (C and D), and for the NETB chains (E and F). The unit of the color scale is 10^–5^. The plots were made with GRI.^48^

Because the multitorsional potential of an ETB chain segment depends on the sum of γ angles along the segment (eq 4), it can be expected that, for an ETB chain segment with length *m* = 5 (i.e., with two consecutive γ angles), the distribution of these angles is narrower along *Δγ*_*N*_ = *Δγ*_*C*_ and broader along *Δγ*_*N*_ = −*Δγ*_*C*_ direction, where *Δγ* is the displacement of the respective angle from distribution center. The reason for this is that, if the changes of the two angles are opposite to each other, the sum of the angles remains constant and, consequently, there is no free-energy cost due to the multitorsional term expressed by eq 4. The respective plots for the ETB chain segments are shown in Figure 5, parts A and C, for θ_*ext*_ = 120° and 135°, respectively. It can be seen from Figure 5, parts A and C, that the γ_*N*_ and γ_*C*_ angles are indeed anticorrelated, the anticorrelation being more pronounced for θ_*ext*_ = 135°. The bulk of the distribution is centered at about γ_*N*_ = 20°, γ_*C*_ = −110°. The anticorrelation between the γ_*N*_ and γ_*C*_ angles is even more apparent from the respective covariance plots shown in panels B and D of Figure 5. The anticorrelation also results in keeping approximately the same dihedral between the two virtual bonds at the end of the segment and the extended-segment axis (the angle Γ′ shown in Figure 2A). There also is another distribution center at about γ_*N*_ = 110°, γ_*C*_ = −120°, which corresponds to uncorrelated angles. The intensity of the two centers is swapped when θ_*ext*_ is smaller (Figure 5A). This is understandable, because the weight of the multitorsional term quickly drops with the interchain-segment θ angle(s) becoming smaller (Figure 4).

It should be noted that the zero distribution of γ_*N*_ and γ_*C*_ is uniform. For a 5-bead polymer chain γ_*N*_ and γ_*C*_ are sampled independently from a uniform distribution and this sampling is independent of the virtual-bond angles θ if the fine-grained degrees of freedom and the interaction energies are not taken into account.

For reference, the distribution and covariance of the γ_*N*_ and γ_*C*_ angles of the NETB chains are shown in Figure 5, parts E and F, respectively. It can be seen that both the distribution and the covariance are different from those shown in panels A–D of the figure, with two peaks at γ_*N*_ of about 50° and γ_*C*_ of about −120° and 40°, respectively, with no correlation between the angles exhibited. It can, therefore, be concluded that the correlation observed in panels B and D of the figure results from extended central θ angle of the 5-residue ETB chain segments.

**Figure 6:**
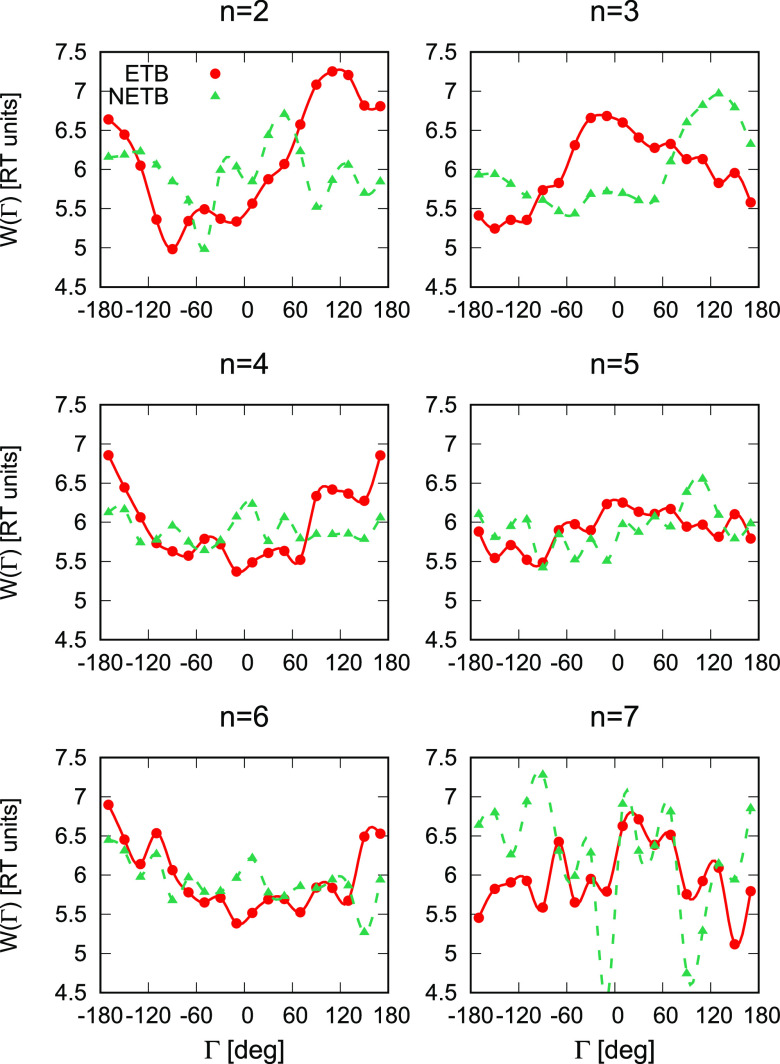
Potentials of mean force in the sum of virtual-bond-dihedral angles γ (Γ) along chain segments with *n* consecutive backbone-virtual-bond dihedrals for the ETB segments of protein chains (filled red circles and solid red lines) and NETB segments of protein chains (filled green triangles and green dashed lines). The lines are the C-splines linking the points. The plot was made with gnuplot.^49^

Because of the insufficient number of data points and increasing dimensionality of the space of the variables, it does not seem meaningful to try to detect the interangle correlations for longer ETB chain segments. However, we can compare the potentials of mean force in the sum of angles (Γ) for the ETB and NETB chains. Sufficient data are available for 5 ≤ *m* ≤ 10 (or the number of dihedrals, 2 ≤ *n* ≤ 7). The respective plots, obtained with θ_*cut*_ = 120°, are shown in Figure 6. The plots for ETB chains with θ_*cut*_ = 135° are similar but could be made only for *n* ≤ 4 because of insufficient statistics. It can be seen from the figure that the PMFs for the ETB chain segments are different from those of the NETB ones. The ETB PMFs exhibit a greater span than those of NETB chain segments, while those for NETB segments exhibit a more pronounced random-noise pattern. It can also be seen that the ETB PMFs are low for smaller γ angles for *n* = 2 and *n* = 4, while for *n* = 3 this region becomes unfavorable. This trend (low PMF for small angles for even *n* and higher for odd *n*) seems to persist for *n* > 4; however, the statistics are poorer in such cases. Therefore, the conclusion from analyzing the γ_*N*_, γ_*C*_ distribution for *n* = 2 (Figure 5) that the dihedral angle Γ′ defined by the two flanking backbone virtual bonds and the extended segment axis (Figure 2A) is largely restricted (being small for an even number of dihedrals and extended for an odd number of dihedrals) seems to extend at least until *n* = 4, although this trend seems to vanish as the length of the extended segment increases.

### Folded Chain Segments

**Figure 7:**
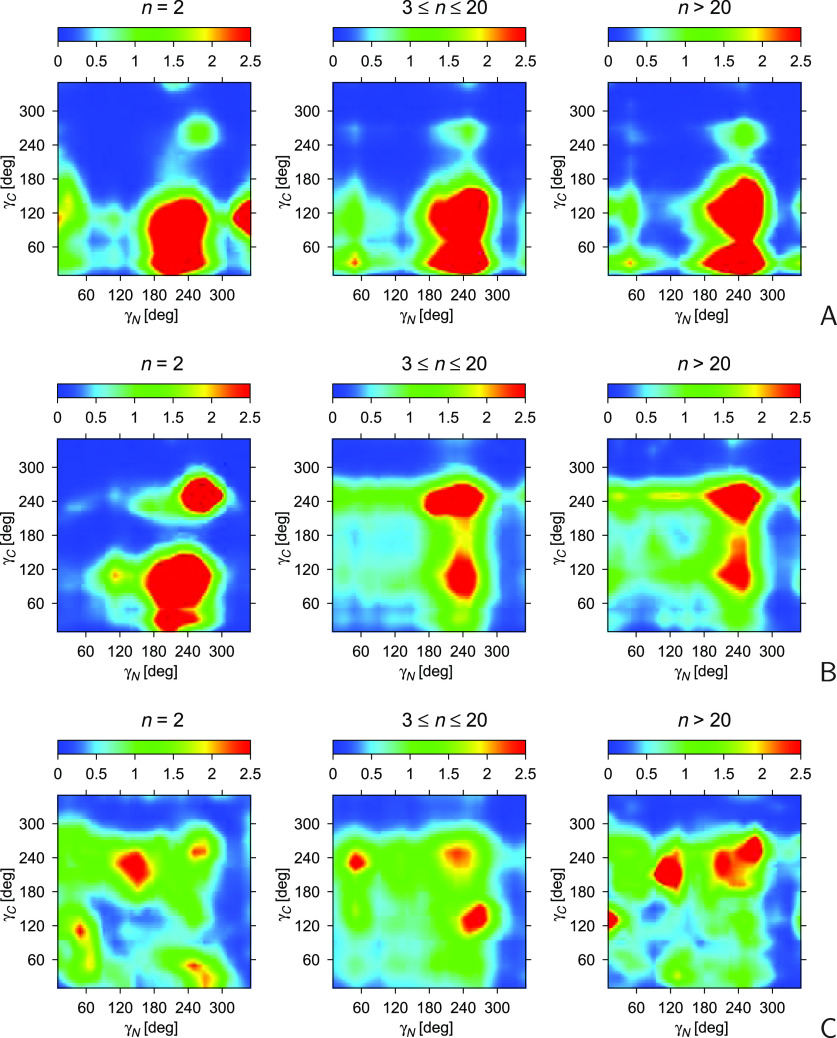
Heat maps of the 2D-distributions (expressed as probability per degree^2^) of the γ_*N*_ and γ_*C*_ for (A) folded (FD), (B) folded helical (FH), and (C) nonstructured (NS) segments of protein chains for three ranges of the number of dihedrals (*n*) contained in a segment. The unit of the color scale is 10^–5^. The plots were made with GRI.^48^

As follows from eq 6, the multitorsional potentials for folded-chain segments depend on the products of the cosines of phase-shifted torsionals along a segment. Therefore, unlike the case of the ETB segments, no correlation between the consecutive dihedrals can be expected. Because the majority of longer folded chain segments are helices, the internal dihedrals are quite restricted in value (to about 45°) and the analysis can be restricted to the distribution of the γ_*N*_ and γ_*C*_ angles. The heat maps of the distributions of (γ_*N*_, γ_*C*_), made for *n* = 2, 3 ≤ *n* ≤ 20, and *n* > 20, are shown in Figure 7A. For comparison, the angles for the nonstructured segments (NS) of the same length range are shown in Figure 7C. To avoid splitting the distribution maxima, both γ_*N*_ and γ_*C*_ range from 0 to 360° in both figures.

It can be seen from Figure 7A that the N-terminal γ angle is quite restricted, being centered around −120° (240°). Conversely, the distribution in γ_*C*_ has one maximum at around 120°, another one at 30°, a less pronounced one at 240° (−120°), and a very narrow one at small γ_*N*_ and γ_*C*_ (the left bottom corner of the plot). The distribution becomes more focused as the length of the folded segment of the chain increases. Because the secondary structures characteristic of folded chain segments are mainly α-helices, we also made plots for the α-helical (FH) segments (Figure 7B). It can be seen that the maximum at γ_*N*_ ≈ 240°, γ_*C*_ ≈ 120° remains a strong one; however, that at γ_*N*_ ≈ γ_*C*_ ≈ 240° becomes the strongest one, and that at γ_*N*_ ≈ 240°, γ_*C*_ ≈ 30° disappears. It should be noted that the analysis of FH chain segments is only an addition to the analysis of general FD chain segments, the definition of which does not depend on secondary-structure assignment but solely on the virtual-bond geometry.

As can be seen from Figure 7C, the distributions of (γ_*N*_, γ_*C*_) of the nonstructured segment of the chain share the maximum at (γ_*N*_ ≈ 120°, γ_*C*_ = 240°). However, the distribution is very diffuse. It should be noted that, for *n* = 2, the plots for the NS chain segments are similar to that of the distribution of two consecutive γ angles reported earlier by Dewitte and Shaknhnovich.^32^

The results obtained for the folded segments of polypeptide chains suggest that the presence of an intervening folded chain segment changes the picture to shift part of the distribution in γ_*C*_ from 60° to 120° and to eliminate most of positive γ_*N*_ dihedrals. The two angles become additionally restricted when a folded segment is α-helical (Figure 7B). Therefore, the folded segment of a chain seems to rigidify both ends, thus largely setting directions to the preceding one and following that segment.

**Figure 8:**
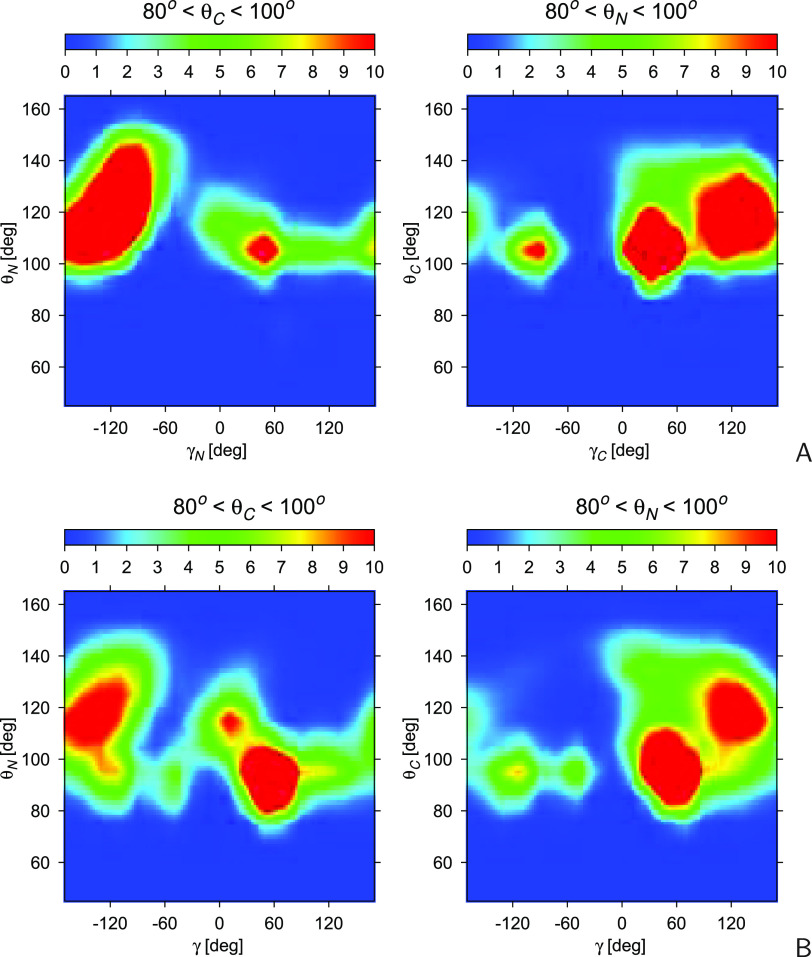
Heat maps of the 2D-distributions (expressed as probability per degree^2^) of γ_*N*_ and θ_*N*_ and those for γ_*C*_ and θ_*C*_ for (A) folded (FD) and (B) nonstructured (NS) segments of protein chains. The unit of the color scale is 10^–5^. The plots were made with GRI.^48^

Because no restrictions were imposed on the terminal θ angles, we also made plots of 2D-distributions of γ_*N*_ and θ_*N*_ and those of γ_*C*_ and θ_*C*_ to determine if the presence of the folded interior changes the angles θ. These distributions for the folded and nonstructured segments of the chains are shown in Figures 8, parts A and B, respectively. As shown, the patterns are similar, and the only differences are in the intensity of the maxima, this arising from the difference of γ-angle populations for the two samples (cf. Figure 7). Therefore, the along-chain interactions seem to have no direct effect on the distribution of the backbone-virtual-bond angles.

### Sequence Dependence

In the considerations presented so far, the amino acid sequence was ignored. However, glycine and proline, which have patterns of local interactions distinctively different from those of other residues, are overrepresented at the ends of helices and strands, which can be seen in Figure S1 of the Supporting Information. We, therefore, made the analysis reported in sections “Extended turn-bordered chain segments” and “Folded chain segments” for reduced data sets, from which all chain segments containing glycine or proline residues were removed. The distributions and the PMFs are shown in Figures S2–S4 of the Supporting Information. As can be seen from these figures, there are no qualitative differences between the plots shown in Figure 5–7 and those shown in Figures S2–S4. Due to reducing the number of data points upon the elimination of the entries with the proline and the glycine residues, the plots of the multitorsional potentials shown in Figure S3 for *n* > 4 are more rugged compared to those of Figure 6, and most of the heat map of the (γ_*N*_, γ_*C*_) distribution for the nonstructured segments with *n* > 20 (Figure S4C) shows zero population, because there are very few unstructured chain segments with length greater than 20 and no glycine or proline residues.

### Use of the Derived Multitorsional Potentials in Coarse-Grained Force Fields and Their Significance

Based on eqs 4 and 6, expressions given by eqs 16–18 can be proposed for the multitorsional potential of an *m*-residue backbone segment starting at residue *i*.
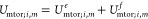
16
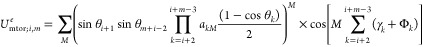
17
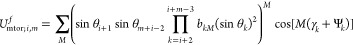
18where *M* is the multiplicity of a given term and the constants *a*_*kM*_ and *b*_*kM*_ and the phase angles Φ_*k*_ and Ψ_*k*_, *k* = *i* + 2, *i* + 3, ... *i* + *m* – 3, are adjustable force-field parameters that depend on the types of residues that are on the axes of the consecutive virtual-bond-dihedral angles of a given segment. Similarly as for single torsional potentials,^16,33^ these constants can be expressed by quantities dependent on single-residue type.

The *U*_*mtor;i,m*_^*e*^ term in eq 16, which is given by eq 17 accounts for the extended-chain segments and becomes unimportant for nonextended chain segments, for which the inner θ angles are much smaller than 180° and, consequently, the factors (1 – cos θ) /2 are much smaller than 1. On the other hand, the term will also become smaller when the first and the last virtual-bond-dihedral angles differ significantly from 90°. Therefore, it will be the most significant for the extended turn-bordered chain segments, which have clearly defined ends. As discussed in the section “Extended turn-bordered chain segments”, the *U*_*mtor;i,m*_^*e*^ term will both make the given relative orientation of the ends of the segment preferable and favor correlated changes of the angles γ along the segment to keep their sum constant.

The *U*_*mtor;i,m*_^*f*^ term (eq 18) accounts for directing the ends of folded (usually α-helical) segments (section “Folded chain segments”), which is significant because this feature could help to achieve the correct chirality of helical bundles. The (sin θ)^2^ terms quickly tend to zero when the angles θ divert from 90°. It should be noted that, as opposed to the *U*_*mtor;i,m*_^*e*^ term, the sine factors do not come from the parent cumulant expression (eq 3) but were introduced to make *U*_*mtor;i,m*_^*f*^ significant only when the inner θ angles are close to 90°. However, the introduction of the sine factors is justifiable because the underlying assumption that allows us to sum all the terms in eq 3 with equal weights (and, thereby, to obtain the expression with the product of the phase-shifted cosines of the consecutive γ angles) is that the inner θ angles of the respective chain segment are close to 90°. Any other unimodal function of θ with a maximum at θ = 90° could also be introduced.

The complete multitorsional term is a sum over all continuous chain segments with different lengths, as given by eq 19.
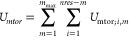
19Here *m*_*max*_ is the maximum assumed backbone-coupling length, which can be different for the folded and for the extended terms, and *nres* is the number of residues in the chain. *m*_max_ another adjustable parameter, which will have to be selected in the process of force-field parametrization.

To check if the absence of multitorsional terms in the present UNRES force field is manifested in the UNRES-modeled protein structures, we constructed and analyzed the two-dimensional distribution and covariance plots of the γ_*N*_ and γ_*C*_ angles for 5-residue ETB chain segments and the distribution plots for the folded and folded helical chain segments for the two series of UNRES-based models in the CASP14 experiment.^24^ The first series contained 260 models from the UNRES group, which did not use any knowledge-based information except for weak restraints on secondary structures from PSIPRED.^34^ The second series contained 290 models from the UNRES-template group, which used restraints from the consensus segments of server models (which were available 72 h after a target had been released) selected on the basis of quality assessment, as described in our earlier work.^35,36^ It should be noted that the restraints in the UNRES-template group were not very strong and were multimodal to cover segments from multiple models. Also, except for strongly homologous targets, the restraints did not cover the whole sequence. All models were in all-atom representation obtained from the UNRES representation by applying the conversion procedure,^23^ which is based on the PULCHRA^37^ and SCWRL^38^ algorithms. The results are shown in Figures S5 and S6 of the Supporting Information, respectively.

It can be seen from Figure S5, parts A and C, that the distribution of the γ_*N*_ and γ_*C*_ angles for the ETB chain segments from the UNRES group models are significantly different from those encountered in proteins. There is a maximum at small positive γ angles, which is virtually not present in protein structures (Figure 5, parts A and C), for which γ_*C*_ is extended and negative. This feature of the NETB chain segments from the UNRES models is especially visible in Figure S5A, in which the distribution for θ_*cut*_ = 120° is shown and for which the maximum at small positive γ dihedral angles becomes the global maximum. It can be noted that the γ angles are anticorrelated for this maximum (Figure S5, parts A and B). However, in this case, the anticorrelation results from the repulsion of the side chains and peptide groups at the termini of the chain segment. If the central θ angle is not so extended (which happens for θ_*cut*_ = 120°, the end groups will overlap unless the end virtual bonds move synchronously. It can be seen from panels C and D of Figure S5 that the anticorrelation disappears for θ_*cut*_ = 135°, for which the end groups do not overlap significantly even when the end backbone virtual bonds of the segment face each other. The regions of the other distribution maxima do not exhibit any anticorrelation of the γ_*N*_ and γ_*C*_ angles.

It can also be seen from Figure S5, parts E and G that the segments from the UNRES-template group models better reflect the distributions derived from protein structures (Figure 5, parts A and C) than those from the UNRES group models. However, a significant distribution maximum is still observed for small γ angles (Figure S5, parts E and G). Moreover, there is no anticorrelation between the γ_*N*_ and γ_*C*_ angles (Figure S5, parts F and H). Therefore, including the information from templates in protein-structure modeling with UNRES seems to help in achieving the correct relative orientation of segment ends but does not seem to capture the concerted change of the γ angles.

The distributions of the γ_*N*_ and γ_*C*_ angles for the FD and FH chains shown in Figure S6, parts A and B, for the UNRES group models and in Figure S6, parts C and D, for the UNRES-template group models, respectively, indicate similar differences from those derived from the PDB as in the case of ETB chain segments. For the UNRES group models, the dominant maxima occur for small positive γ_*C*_, unlike the distributions from the PDB (Figure 7), in which γ_*C*_ is more extended and also achieves values greater than 180° (or negative) for the folded helical (FH) segments. Moreover, for the UNRES group models, there is also a strong maximum of the distribution for small γ_*N*_, which is only very weak in protein structures. Thus, neglecting the long-range backbone correlations is likely to lead to problems with achieving correct helix topology in UNRES modeling. As can be seen from Figure S6C and S6D, the distributions obtained from the UNRES-template group models are closer to those from the PDB.

From the above analysis of the distributions of angles from the UNRES-based models it appears that the UNRES force field, and probably other coarse-grained force fields and all-atom force fields, can benefit from introducing the terms accounting for the correlation between backbone conformational states, which are expressed as multitorsional potentials. These terms can help in finding the correct orientation of helices and strands, which is crucial in correct packing of those elements and, consequently, in protein-structure modeling in the chemical or *ab initio* mode. It also appears that at least some part of the correlations is captured by bioinformatics-based methods.

### Role of the Along-Chain Correlations in Shaping Protein Structures and in Relaying Conformational Changes

The results presented in the sections “Extended turn-bordered chain segments” and “Folded chain segments” strongly suggest that the coupling between the local conformational states along the extended and folded segments results in restricting the available orientations of the end virtual bonds of a given segment. For the folded segments (which are mostly helical), each of the end bonds is fixed independently. Therefore, such a segment acts as a “vise” (with two pairs of jaws) fixing the ends. On the other hand, an extended segment only fixes the relative orientation of its ends, because the respective potential of mean force depends on the sum of the angles γ along the chain (eq 4), which is approximately equal to the dihedral angle Γ′ (Figure 2A) formed by the end virtual bonds and the segment axis. There also is a difference in the range of the fixing effect; it persists for any length of a folded segment (Figure 7, parts A and B), while it remarkably diminishes with increasing segment length for extended segments (Figure 6).

The cooperativity of local interactions along a chain segment seems to be important in orienting secondary-structure elements with respect to each other and, in turn, probably is one of the important factors determining the packing of helices and strands. It seems worthwhile to check if such or similar correlations have been detected by the AI of AlphaFold2 and added to its “dictionary” and “grammar rules” of predicting protein structures. It would also be worthwhile to see if the effect of correlated mutations of the residues that do not make a contact in a native protein can be explained in terms of long-range correlations between local conformational states.

The multitorsional potential derived for the extended turn-bordered (ETB) chain segments does not change when the sum of virtual-bond-dihedral angles along the segment remains constant. This means that changing the orientation of one end virtual bond can be accomplished at a reduced free-energy cost if it is reciprocated by the change at the other end. The coupling of local conformational states found in our work for extended chain segments is also similar to the dark-soliton-like modes found by Niemi and co-workers^29,30^ by applying the Discrete Nonlinear Schrödinger equation to coarse-grained polypeptide chains.

It should be noted that the change of the orientation of a backbone virtual bond at a given end can be induced by the change of the state of the side chain attached to it, e.g., by ligand binding. Such a correlated change of conformational states at two sites, which do not make a contact is characteristic of allosteric interactions. The concept of allosteric interactions was originally restricted to indirect interactions between different chains of multichain proteins such as, e.g., hemoglobin;^39,40^ however, it turned out to be an intrinsic property of all proteins.^41^ One of the present views of allostery is that the change of the distribution of conformational states at one site induces that at the other site.^42,43^ The mechanism of how the change is relayed has been studied by molecular dynamics.^44,45^ Recently Zhu and co-workers^46^ combined molecular dynamics with neural network analysis to find the connection networks. These studies were focused on finding the networks of interacting side chains. From our study, it follows that adding the backbone to the considerations could be beneficial in understanding allosteric interactions. It should be noted at this point that the anticorrelation of the consecutive backbone virtual-bond-dihedral angles γ of extended chain segments shown in Figure 5A–D does not demonstrate allostery as such, because allostery is a causal-effectual phenomenon. However, it strongly suggests that the correlation contribution to the local component of the potential of mean force given by eq 4 provides a smooth road for allosteric interactions to occur.

Further to the above considerations, a question could be asked as to whether the anticorrelation pattern of the consecutive γ angles in allosteric proteins is different from that of the whole set of proteins studied. In an attempt to answer this question, we selected from the set of proteins considered in this study (see section “Protein structure analysis”) those whose PDB files contained the “ALLO” keyword. The selected proteins should thus exhibit or be related to the allosteric behavior. The selected subset contained 1,101 entries, which are collected in File S2 of the Supporting Information. To make a fair comparison, we also created three other sets, each with 1,101 entries selected at random. These entries are collected in Files S3–S5 of the Supporting Information. Subsequently, we calculated the distributions and correlations of two consecutive γ angles of 5-residue ETB segments, taking θ_*cut*_ = 135°. The respective 2D plots are shown in Figure S7 of the Supporting Information. The limited size of the data sets did not enable us to derive and compare the statistical multitorsional potentials (cf. Figure 6).

It can be seen from panels C–H of Figure S7 that the plots corresponding to the randomly selected subsets of proteins do not differ significantly from those of the whole protein set (Figure 5, parts A and B) and do not differ significantly from each other. In contrast to this, those of the proteins that are involved in allostery do. The region of the main distribution maximum (centered at about γ_*N*_ = 10°, γ_*C*_ = −110° for the whole set of proteins) is shifted toward more negative γ_*C*_ angles and the region of the adjacent distribution maximum, which appears at large positive γ_*N*_ angles shows anticorrelation between γ_*N*_ and γ_*C*_, which does not occur in the distributions derived from the entire set of proteins (Figure 5, parts A and B) or from the randomly selected subsets (panels C–H of Figure S7). This result suggests that the anticorrelation between the consecutive backbone-virtual-bond dihedrals is more pronounced for allosteric proteins than for randomly picked proteins. Consequently, it seems that allosteric interactions could be relayed by at least 5-residue segments of protein backbone. However, as stated above, the observed anticorrelations are not identical with allostery as such.

An important finding of the conditions for conformational-change propagation along the protein backbone found in this work is that it occurs along extended backbone segments. Thus an ETB backbone segment acts as a straight piece of wire with two ends bent (similar to a simple lock-pick); displacing one bent end involves a reciprocal displacement of the other one. This concept can be generalized to concerted changes at larger coarse-graining level. For example, in the SURPASS coarse-grained model of proteins developed by Dawid and co-workers,^47^ an α-helix turn is a coarse-grained particle, and as a result, the α-helical segments form nearly straight lines. Thus, the correlated changes could also be propagated along helical segments. Moreover, the results can also be generalized to networks of interacting side chains forming allosteric-interaction pathways. The noncovalent side chain–side chain interactions connecting the side chains would then play a role of covalent backbone interactions. It has been suggested by other researchers^46^ that the interacting side chains should form the shortest (presumably a straight) path for the allosteric interactions to be effective.

## Conclusions

The results of the analysis of the backbone-virtual-bond dihedral angles of the extended turn-bordered and the folded segments of polpyeptide chains suggest that there is a large tendency to determine the directions of the chain parts preceding and following such segments. Thus, not only the long-range interactions but also the sequence of local interactions seems to be an important factor determining the packing of secondary-structure elements and, consequently, shaping protein structure. As mentioned in the section “Role of the Along-Chain Correlations in Shaping Protein Structures and in Relaying Conformational Changes”, it seems worth to check if AlphaFold detects such along-chain correlations on the way of predicting protein structure and if such correlations are manifested in the effect of correlated mutations in which the mutated residues do not make a contact.

For the extended turn-bordered segments (strands), the driving force has a form of the potential dependent on the sum of the virtual-bond dihedrals along the segment, which is approximately equal to the dihedral angle Γ′ (Figure 2A) formed by the end virtual bonds and the segment axis. Its effect is to restrict the relative orientation of one end with respect to the other one. A folded segment (usually a helix) restricts the mobility of the virtual bonds preceding and succeeding the chain, each one independently. The respective multitorsional term (eq 6) does not show that the changes at one end of the chain are relayed to those at the other end and the distributions of the γ_*N*_ and γ_*C*_ angles do not show it either (Figure 7). As shown in the section “Use of the Derived Multitorsional Potentials in Coarse-Grained Force Fields and Their Significance”, with the example of the structures obtained with the UNRES coarse-grained model of polypeptide chains,^19−22^ the sequential correlations between local interactions are likely to improve the performance of coarse-grained force fields by promoting correct orientation of secondary-structure elements. We proposed tentative expressions for the respective coupling terms (eqs 16–18), and work is now in progress in our laboratory to implement them in UNRES and to parametrize them.

As discussed in the section “Role of the Along-Chain Correlations in Shaping Protein Structures and in Relaying Conformational Changes”, the reduced free-energy cost of the concerted rotation about the backbone virtual bonds is likely to contribute to allosteric interactions. This is in agreement with the view of allosteric networks as composed of the shortest connections between the units that relay a conformational change.^42,43,46^ In this regard, it seems to be possible to employ the mathematical formalism developed in this work to identify allosteric networks composed of noncovalently interacting elements (e.g., the side chains) or propagating through helical segments, which can be coarse-grained to nearly linear segments by applying the model of Dawid and co-workers, in which a turn of a helix is a unit.^47^ Research in these directions is planned in our laboratory. The advantage of such an approach is that it enables us to find potential allosteric-interaction networks based on the features of protein structures, without the necessity of doing molecular-dynamics simulations and analyzing their results. It should be noted, though, that such an approach will only identify the possible and not the actual allosteric pathways.

Another important point is that fixing the ends of the extended or folded chain segments and reduced free-energy cost of concerted rotations about the virtual bond of extended chain segments (cf. eqs 4 and 6) found in this work do not depend on the details of the all-atom geometries of the units or those of the parent all-atom energy surfaces, which are hidden in the coefficients and in the phase angles. These features arise exclusively from expressing the distance between the two atoms of the consecutive units in terms of the geometry of their virtual-bond axes, their location in the local-coordinate systems of these units, and the angles for the rotation about the virtual bonds (cf. eq 35 and Figure 2 in ref (16)). In other words, these “along-chain-correlation” terms are a consequence of the fact that protein chains are embedded in the three-dimensional Euclidean space. On the other hand, the directions of chain-end orientation and the extent of the orientation effect certainly depend both on valence-geometry details and on the local-interaction pattern.

## Supporting Information Available

Derivation of the lowest-order term in multitorsional potentials; Figure S1, histograms of the fractions of specific residue types in the first and second position at the N- and at the C-terminus of (A) ETB and (B) F chains; Figure S2, heat maps of the 2D-distribution of γ_*N*_ and γ_*C*_ for ETB chain segments for (A) θ_*cut*_ = 120°, (B) θ_*cut*_ = 135° and (C) NETB chain segments obtained after eliminating the entries with the glycine and proline residues at the ends of the segments; Figure S3, potentials of mean force in the sum of virtual-bond-dihedral angles γ(Γ) along chain segments with *n* consecutive backbone-virtual-bond dihedrals for the ETB and NETB segments of protein chains obtained after eliminating the entries with the glycine and proline residues at the ends of the segments; Figure S4, heat maps of the 2D-distributions of γ_*N*_ and γ_*C*_ for (A) FD, (B) FH, and (C) NS chains obtained after eliminating the entries with the glycine and proline residues at the ends of the segments; Figure S5, heat maps of the 2D-distributions and correlations of γ_*N*_ and γ_*C*_ for 5-residue ETB segments from the CASP14 UNRES group models and the CASP14 UNRES-template models; Figure S6, heat maps of the 2D-distributions of the γ_*N*_ and γ_*C*_ corresponding to the CASP14 UNRES group models and CASP14 UNRES-template groups models; Figure S7, heat maps of the 2D-distributions and correlations of γ_*N*_ and γ_*C*_ for 5-residue ETB segments from 1101 allosteric proteins and three sets of 1101 proteins each, selected at random from the PDB (PDF)
File S1: List of all proteins chains analyzed in this study (TXT)
File S2: List of allosteric proteins analyzed in this study (TXT)
File S3: Set 1 of the three randomly picked sets of 1,101 proteins each analyzed in this study (TXT)
File S4: Set 2 of the three randomly picked sets of 1,101 proteins each analyzed in this study (TXT)
File S5: Set 3 of the three randomly picked sets of 1,101 proteins each analyzed in this study (TXT)
