# Correction to “Origin of Correlations between Local Conformational States of Consecutive Amino-Acid Residues and Their Role in Shaping Protein Structures and in Allostery”

**DOI:** 10.1021/acs.jpcb.2c08574

**Published:** 2022-12-22

**Authors:** Celina Sikorska, Adam Liwo

The authors regret that errors were made in the derivation of eq 3C, which also affect the final form of eq 6C but not that of eq 4. These errors do not change the conclusions of the paper, because the corrected eq 6C still expresses a multitorsional potential that is a product of cosines of virtual-bond dihedrals along a folded chain segment except that there are sines and not cosines of the first and the last dihedral, respectively, while cosines only appeared in the incorrect equation. Thus, the corrected expression still corresponds to directing the chain before and after a folded (in most cases a helical) chain segment.

The corrected eqs 3C and 6C are below. To keep correspondence with the original paper, they are labeled 3C and 6C, respectively. The revised derivation of both equations is provided in the Supporting Information.
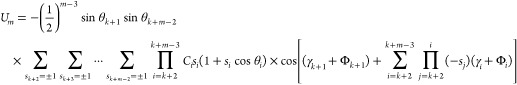
3C
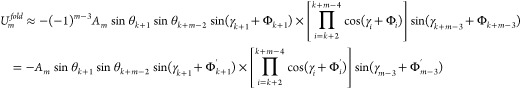
6Cwhere
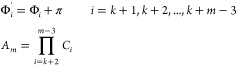


In eqs 3C and 6C, *m* is the number of C^α^ atoms in the segment (the length of the segment), *k* is the index of the first residue of the segment, θ_*i*_ is the planar angle between , , and , and γ_*i*_ is the dihedral angle defined by atoms , , , and . The angles Φ_*i*_ and  are phase angles and the coefficients *C*_*i*_ depend on the kind of respective amino-acid residues and the neighboring residues.

Following the correction, eq 18C, which expresses the multitorsional energy term corresponding to a folded chain segment, , which we recommend to introduce to coarse-grained force fields, is replaced by eq 18C.
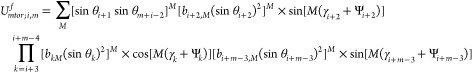
18Cwhere *M* is the multiplicity of the respective term and the coefficients *b*_*i*,*M*_ are parameters.

## Supporting Information Available

Correction to “Derivation of the lowest-order term in multitorsional potentials” and “Derivation of eq 6C” (PDF)

