# Supplementary material for: Origin of Correlations between Local Conformational States of Consecutive Amino Acid Residues and Their Role in Shaping Protein Structures and in Allostery
Source: J Phys Chem B. 2022 Nov 11;126(46):9493–505. doi: 10.1021/acs.jpcb.2c04610 (PMC9706564; doi:10.1021/acs.jpcb.2c04610)
Supplement: Supplementary file 1 — jp2c04610_si_001.pdf [file jp2c04610_si_001.pdf]

Supporting Information
Origin of Correlations between Local Conformational States of Consecutive Amino-Acid Residues and Their Role in Shaping Protein Structures and in Allostery

Celina Sikorska† and Adam Liwo*,‡

†*The MacDiarmid Institute for Advanced Materials and Nanotechnology, Department of Physics, The University of Auckland, Private Bag 92019, Auckland 1142, New Zealand*

‡*Faculty of Chemistry, University of Gdańsk, Fahrenheit Union of Universities in Gdańsk, Wita Stwosza 63, 80-308 Gdańsk, Poland*

E-mail: adam.liwo@ug.edu.pl

Phone: +48585235124. Fax: +48585235012

Derivation of the lowest-order term in multitorsional potentials

Following the methodology presented in ref 1, the lowest-order Kubo cluster cumulant[2] in the expansion of the generic $(m-2)$nd-order contribution (i.e., encompassing the whole chain segment under consideration) to the potential of mean force of the local interactions of an m-residue segment of a polypeptide chain, from C_k^α to C_{k+m-1}^α, is expressed by eq S1.

$$
\begin{aligned}
U_m &= -(-1)^{m-2} \sum_{m_k} \sum_{m_{k+1}} \sum_{m_{k+2}} \cdots \sum_{m_{k+m-3}} \sum_{m_{k+m-2}} \left(\prod_{i=k+2}^{k+m-2} c_{i,m_i} \right) \langle f_{k,m_k;k+1,m_{k+1}} g_{k+1,m_{k+1};k+2,m_{k+2}} \\
&\quad \cdots g_{k+m-4,m_{k+m-4};k+m-3,m_{k+m-3}} f_{k+m-3,m_{k+m-3};k+m-2,m_{k+m-2}} \rangle \qquad \text{(S1)}
\end{aligned}
$$

where the terms $f_{i\alpha;j\beta}(\lambda_i)$, and $g_{i\alpha;j\beta}(\lambda_i,\lambda_j)$ are the terms of the expression of the distance between atom with index α of unit i and that with index β of unit j in the angles λ_i or λ_i and λ_j for the rotation of those atoms about the respective virtual-bond axes, as expressed by eqs 32 – 42 and illustrated in Figure 2 of ref 1, and $\langle \cdots \rangle$ denotes the average over all λs. The coefficients $c_{i,m_i},\ i = 1,2,\ldots,\ k+m-2$ depend on the derivatives of the energy of interactions between the respective atoms on distance (cf eq 43 of ref 1).

Because the fs and the gs correspond to adjacent units, they can be expressed by eqs S2 – S4, respectively (see also eqs 77 – 79 of ref 1).

$$
f_{i\alpha;i+1,\beta}(\lambda_{i+1}) = a_{i\alpha;i+1,\beta} \sin\theta_{i+1} \cos\left(\lambda_{i+1} + \phi_{i+1,\alpha} - \Psi_{i,i+1}\right) \qquad \text{(S2)}
$$

$$
f_{i+1,\alpha;i\beta}(\lambda_i) = a_{i+1,\alpha;i,\beta} \sin\theta_{i+1} \cos\left(\lambda_i + \phi_{i,\alpha} - \Psi_{i+1,i}\right) \qquad \text{(S3)}
$$

$$g_{i\alpha;i+1,\beta}(\lambda_i,\lambda_{i+1}) = b_{i\alpha;i+1,\beta}\Big\{(1+\cos\theta_{i+1})\cos[(\lambda_i+\phi_{i\alpha}-\Psi_{i+1,i})$$
$$+(\lambda_{i+1}+\phi_{i+1,\beta}-\Psi_{i,i+1})]$$
$$-(1-\cos\theta_{i+1})\cos[(\lambda_i+\phi_{i\alpha}-\Psi_{i+1,i})-(\lambda_{i+1}+\phi_{i+1,\beta}-\Psi_{i,i+1})]\Big\} \quad \text{(S4)}$$

where the coefficients $a_{i\alpha;i+1,\beta}$ and $b_{i\alpha;i+1,\beta}$ depend on the location of atoms with local indices α and β with respect to the origin and virtual-bond axis of the unit, $\phi_{i+1,\alpha}$ is the angle of the rotation of atom with local index α of unit i about the virtual-bond axis in the reference configuration (for planar units ϕ is 0 or 180°), and $\Psi_{i+1,i}$ is the angle of the rotation of the virtual-bond axis of unit $i+1$ about the virtual-bond axis of unit i (see Figure 2 in ref 1).

It should be noted that, apart from those of eq S1, expressions corresponding to the m-bead segments of polypeptide chains that contain the f-terms inside the sequence also occur. However, because each of the consecutive f-term inside the sequence depends on a single λ only, these expressions are products of the components of U_m for shorter chain segments, as shown by eq S5 for an example. Therefore, they will get subtracted in the expression for the generic $(m-2)$-nd order cumulant (see eq 15 in ref 1). On the other hand, the products of the multi-torsional terms corresponding to consecutive chain segments could appear in the force-field expressions for multitorsional potentials (see for example eq 90 in ref 1 that expresses double-torsional potentials), because they correspond to higher-order terms in the cumulant expansion for shorter chain segments.

$$\langle f_{k,k+1}g_{k+1,k+2}\cdots g_{l-1,l}f_{l+1,l}f_{l,l+1}g_{l+1,l+2}\cdots g_{k+m-4,k+m-3}f_{k+m-3,k+m-2}\rangle =$$
$$\langle f_{k,k+1}g_{k+1,k+2}\cdots g_{l-1,l}f_{l+1,l}\rangle\langle f_{l,l+1}g_{l+1,l+2}\cdots g_{k+m-4,k+m-3}f_{k+m-3,k+m-2}\rangle \quad \text{(S5)}$$

For clarity sake, the atom indices m_i are dropped in eq S5.

The components of U_m are expressed by eq S6 (for clarity sake, the atom indices m_i are dropped).

$$\langle f_{k,k+1} g_{k+1,k+2} \cdots g_{k+m-4,k+m-3} f_{k+m-3,k+m-2} \rangle = \left(\frac{1}{2\pi}\right)^{m-3} a_{k,k+1} a_{k+m-1,k+m-2} \times \left(\prod_{i=k+2}^{k+m-3} b_i\right) \sin\theta_{k+1} \sin\theta_{k+m-2} \sum_{s_{k+2}=\pm 1} \cdots \sum_{s_{k+m-2}=\pm 1} \prod_{i=k+2}^{k+m-3} s_i (1 + s_i \cos\theta_i) I_m^{s_{k+2},\ldots s_{k+m-3}} \quad \text{(S6)}$$

$$I_m^{s_{k+2},\ldots s_{k+m-3}} = \int_{-\pi}^{\pi} \cdots \int_{-\pi}^{\pi} \cos(\lambda_{k+1} + \phi_{k+1} - \Psi_{k,k+1}) \times \prod_{i=k+2}^{k+m-3} \cos\left[(\lambda_{i-1} + \phi_{i-1} - \Psi_{i,i-1}) + s_i(\lambda_i + \phi_i - \Psi_{i-1,i})\right] \times \cos(\lambda_{k+m-3} + \phi_{k+m-3} - \Psi_{k+m-2,k+m-3}) d\lambda_{k+2} \ldots d\lambda_{k+m-3} \quad \text{(S7)}$$

By using the Euler formula to express the cosines in terms of imaginary exponentials, which reduces the integration to finding all exponential terms with zero coefficients at λs (see, e.g., eqs 81 and 82 in ref 1) , and by making use of the fact that the difference of the Ψ angles of two consecutive units give the virtual-bond dihedral angle γ shifted by 180° (ref 1)

$$\gamma_i = \Psi_{i+1,i} - \Psi_{i-1,i} + \pi \quad \text{(S8)}$$

the integrals of eq S7 are expressed by eq S9.

$$I_m^{s_{k+2},\ldots s_{k+m-3}} = (-\pi)^{m-3} \cos\left[(\gamma_{k+1} + \delta_{k+1}) - \sum_{i=k+2}^{k+m-2} s_i(\gamma_i + \delta_i)\right] \quad \text{(S9)}$$

$$\delta_i = \phi_{i+1} - \phi_i \quad \text{(S10)}$$

Plugging eq S9 into eq S6 and then, after reintroducing atom indices, eq S6 into eq S7

and, finally applying recursively the reduction formulas for cosines of different angles to get the same phase angles in all terms, canceling the $(-1)^{m-3}$ factors, and defining

$$C_i = c_{i,m_i} a_{k,k+1} a_{i+m-1,i+m-2} b_i \tag{S11}$$

we finally obtain eq 3 of the main text.

References

(1) Sieradzan, A. K.; Makowski, M.; Augustynowicz, A.; Liwo, A. A General Method for the Derivation of the Functional Forms of the Effective Energy Terms in Coarse-Grained Energy Functions of Polymers. I. Backbone Potentials of Coarse-Grained Polypeptide Chains. *J. Chem. Phys.* **2017**, *146*, 124106.

(2) Kubo, R. Generalized Cumulant Expansion Method. *J. Phys. Soc. Japan* **1962**, *17*, 1100–1120.

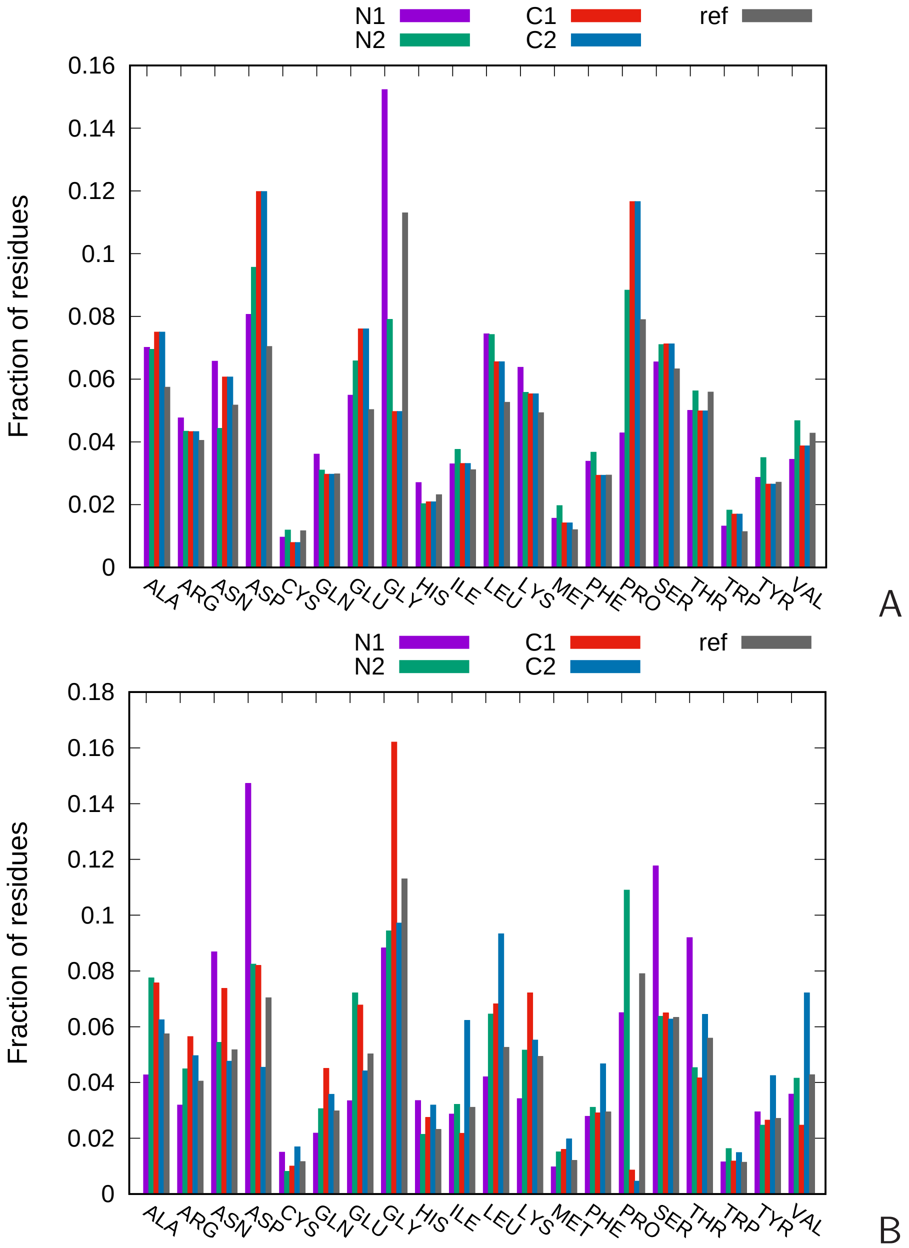

Figure S1: Histograms of the fractions of specific residue types in the first and second position at the N- (N1 and N2) and at the C-terminus (C1 and C2) of (A) ETB and (B) F chains. The reference (ref) bars correspond to the data from the entire set.

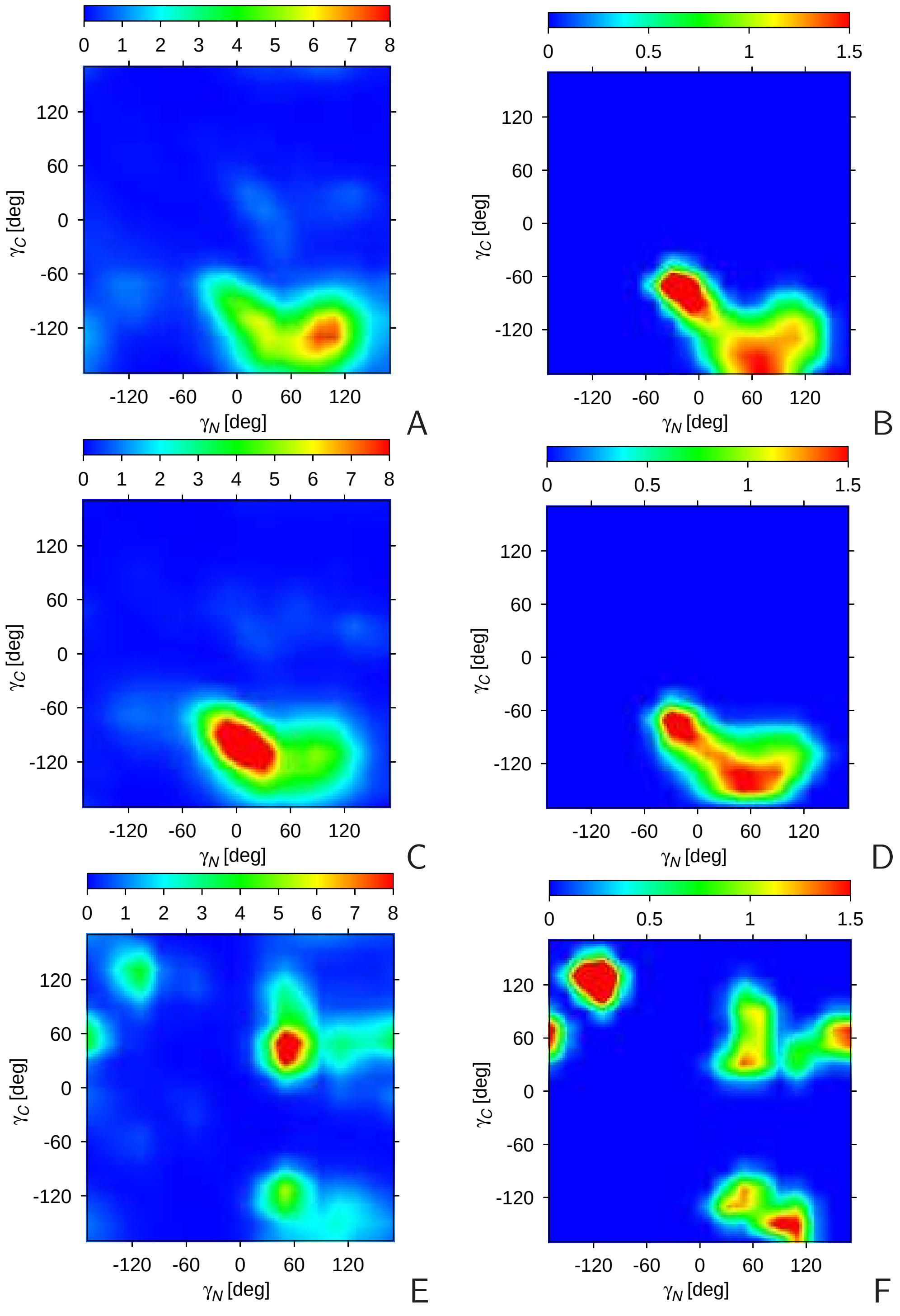

Figure S2: Heat maps of the 2D-distributions (expressed as probability per degree^2) (A, C, and E) and correlations (B, D, and F) of γ_N and γ_C for 5-residue ETB segments of proteins chains not containing glycine or proline residues, derived assuming the cut-off for the central θ angle, $\theta_{cut} = 120°$ (A), $\theta_{cut} = 135°$ (B) and for the NETB segments of protein chains not containing proline or glycine residues (C). The unit of the color scale is 10^{-5} on panels A, C, and E and 1 on panels B, D, and F, respectively. The plots were made with `GRI`.

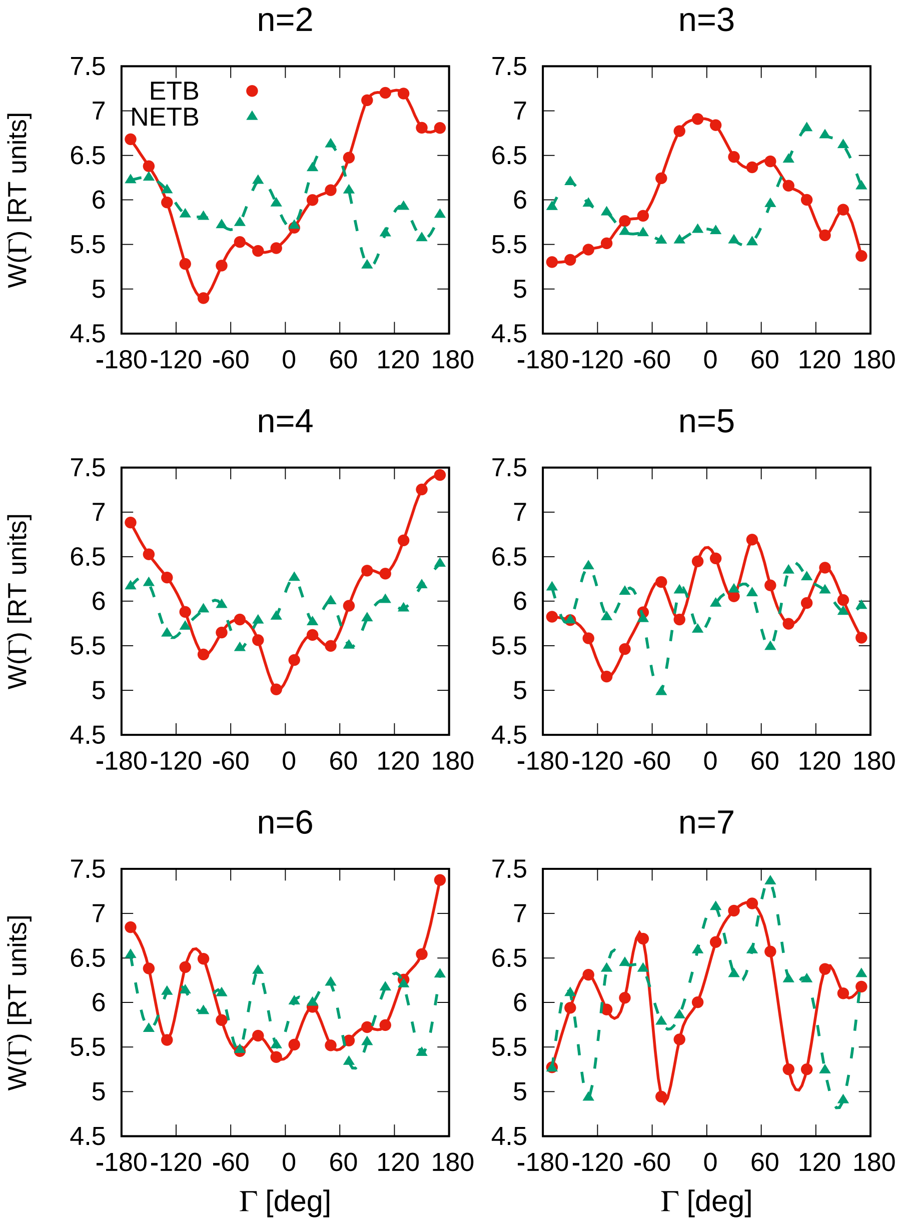

Figure S3: Potentials of mean force in the sum of virtual-bond-dihedral angles γ (Γ) along chain segments with n consecutive backbone-virtual-bond dihedrals for the ETB segments of protein chains (filled red circles and solid red lines) and NETB segments of protein chains (filled green triangles and green dashed lines) not containing glycine or proline residues. The lines are the C-splines linking the points. The plot was made with `gnuplot`.

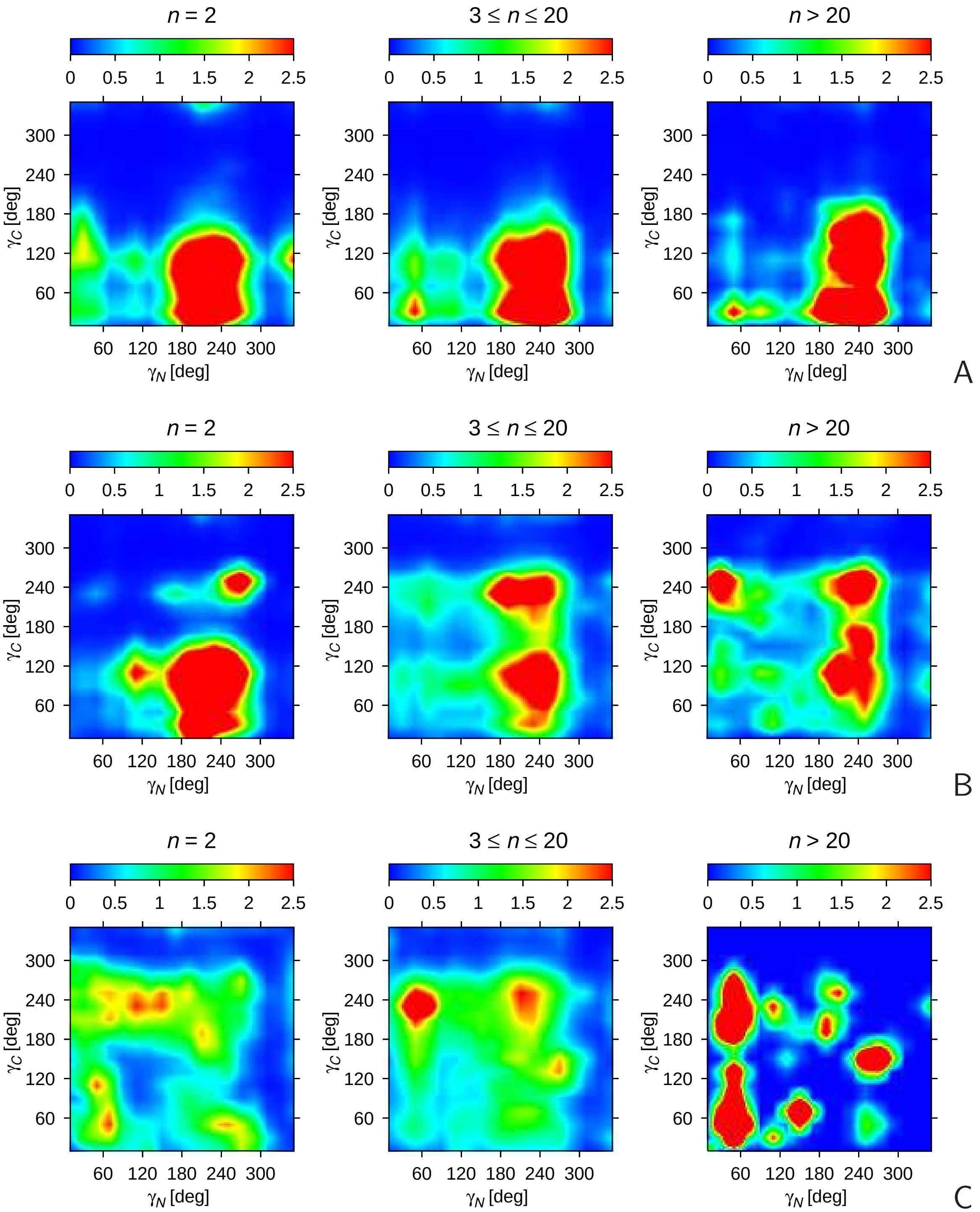

Figure S4: Heat maps of the 2D-distributions (expressed as probability per degree^2) of the γ_N and γ_C for (A) folded (FD), (B) folded helical (FH), and (C) non-structured (NS) segments of protein chains not containing glycine or proline residues for three ranges of the number of dihedrals (n) contained in a segment. The unit of the color scale is 10^{-5}. The plots were made with `GRI`.

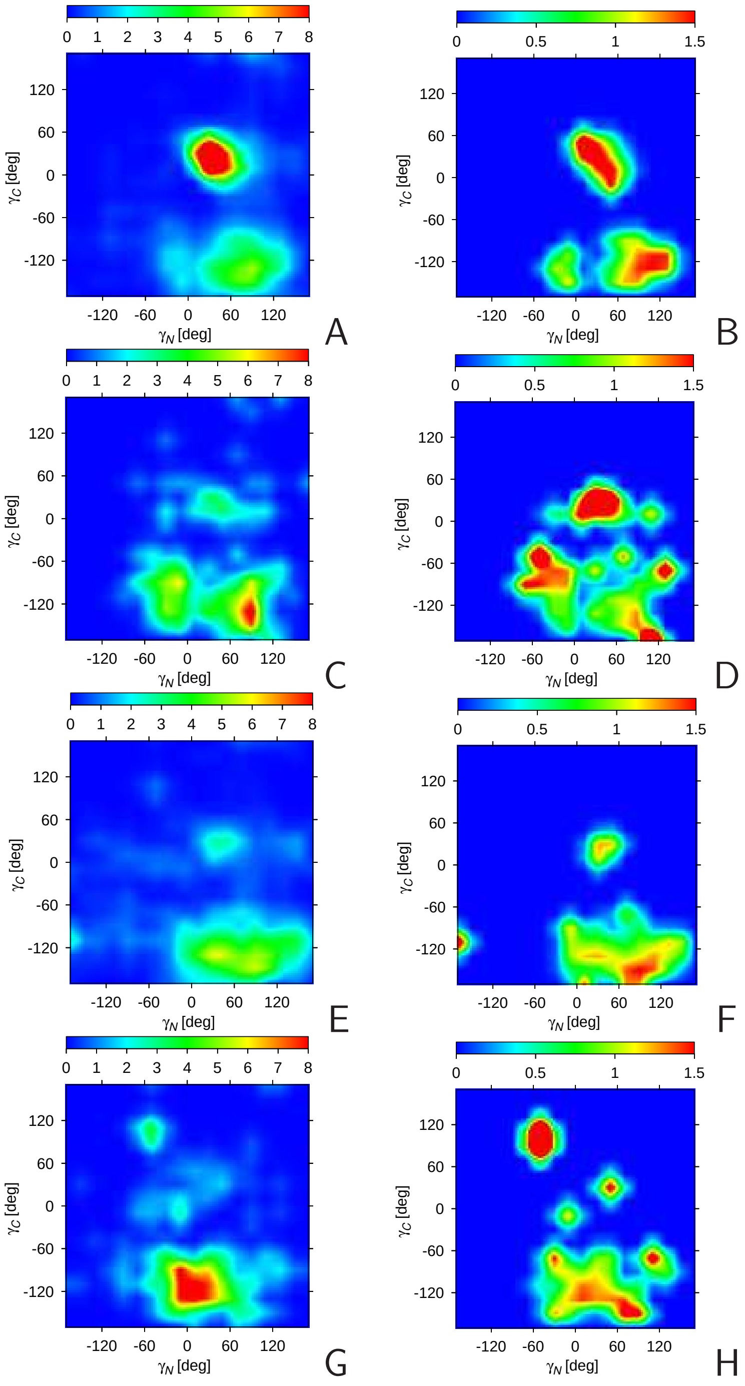

Figure S5: Heat maps of the 2D-distributions (expressed as probability per degree^2) (A, C, E, G) and correlations (B, D, F, H) of γ_N and γ_C for 5-residue ETB segments from the CASP14 UNRES group models (A–D) and the CASP14 UNRES-template models (E-H) derived assuming the cut-off for the central θ angle, $\theta_{cut} = 120^\circ$ (A, B, E, F), $\theta_{cut} = 135^\circ$ (C, D, G, H). The unit of the color scale is 10^{-5} on panels A, C, E, and G and 1 on panels B, D, F, and H, respectively. The plots were made with `GRI`.

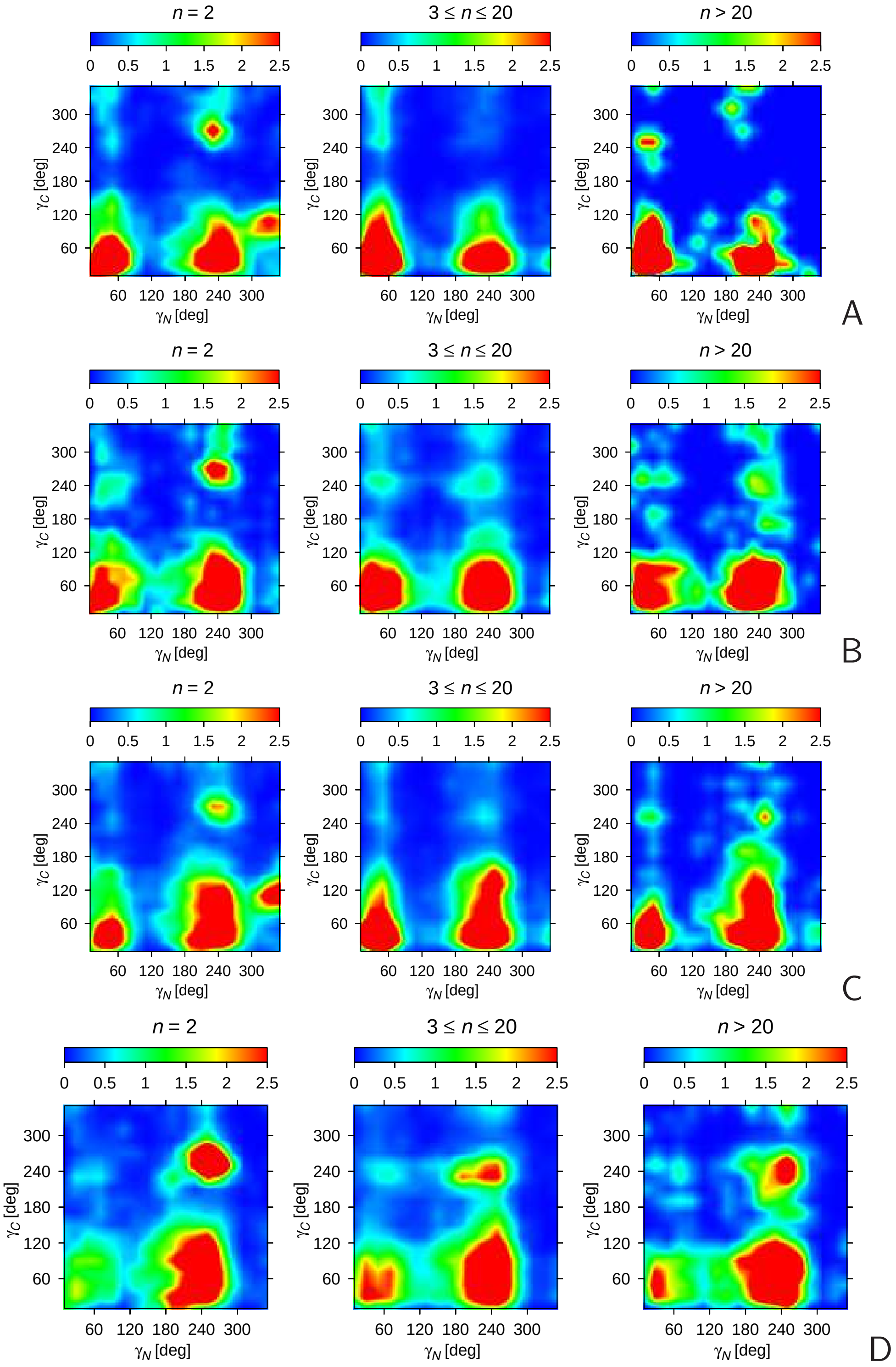

Figure S6: Heat maps of the 2D-distributions (expressed as probability per degree^2) of the γ_N and γ_C corresponding to the CASP14 UNRES group models (A and B) and CASP14 UNRES-template groups models (C and D) for the folded (FD) (A and C) and folded helical (FH) (B and D) chain segments for three ranges of the number of dihedrals (n) contained in a segment. The unit of the color scale is 10^{-5}. The plots were made with `GRI`.

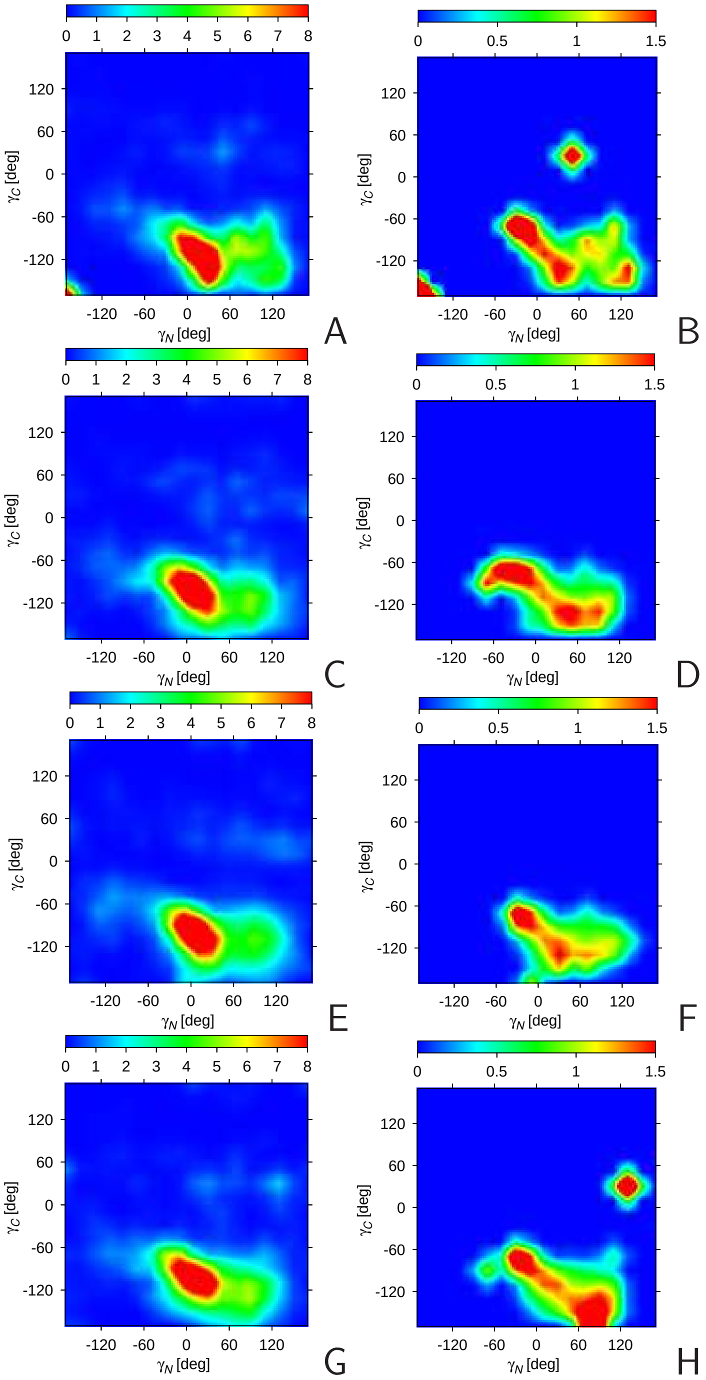

Figure S7: Heat maps of the 2D-distributions (expressed as probability per degree^2) (A, C, E, G) and correlations (B, D, F, H) of γ_N and γ_C for 5-residue ETB segments from 1,101 allosteric proteins (A and B) and three sets of 1,101 proteins each, selected at random from the PDB. The distributions were derived assuming the cut-off for the central θ angle, $\theta_{cut} = 135°$. The unit of the color scale is 10^{-5}. The plots were made with `GRI`.
