# Supplementary material for: Correction to “Origin of Correlations between Local Conformational States of Consecutive Amino-Acid Residues and Their Role in Shaping Protein Structures and in Allostery”
Source: J Phys Chem B. 2022 Dec 22;127(1):425–6. doi: 10.1021/acs.jpcb.2c08574 (PMC9841556; doi:10.1021/acs.jpcb.2c08574)
Supplement: Supplementary file 1 — jp2c08574_si_001.pdf [file jp2c08574_si_001.pdf]

Supporting Information
Correction to "Origin of Correlations between Local Conformational States of Consecutive Amino-Acid Residues and Their Role in Shaping Protein Structures and in Allostery"

Celina Sikorska† and Adam Liwo*,‡

†*The MacDiarmid Institute for Advanced Materials and Nanotechnology, Department of Physics, The University of Auckland, Private Bag 92019, Auckland 1142, New Zealand*

‡*Faculty of Chemistry, University of Gdańsk, Fahrenheit Union of Universities in Gdańsk, Wita Stwosza 63, 80-308 Gdańsk, Poland*

E-mail: adam.liwo@ug.edu.pl

Phone: +48585235124. Fax: +48585235012

Correction to "Derivation of the lowest-order term in multi-torsional potentials"

We recall eqs S1, S6 and S7 of the Supporting Information of the original paper.[1]

$$U_m = -(-1)^{m-2}\sum_{m_k}\sum_{m_{k+1}}\cdots\sum_{m_{k+m-2}}\sum_{m_{k+m-1}}\left(\prod_{i=k+1}^{k+m-3} c_{i,m_i}\right)\langle f_{k,m_k;k+1,m_{k+1}} g_{k+1,m_{k+1};k+2,m_{k+2}} \cdots g_{k+m-4,m_{k+m-4};k+m-3,m_{k+m-3}} f_{k+m-3,m_{k+m-3};k+m-2,m_{k+m-2}}\rangle \quad \text{(S1)}$$

where the m_i is the index of the mth atom of the ith site and c_{i,m_i} is the coefficients of the energy expansion in the distance between atoms m_i of residue i and atom m_{i+1} of residue $i+1$.

$$\langle f_{k,k+1} g_{k+1,k+2} \cdots g_{k+m-4,k+m-3} f_{k+m-3,k+m-2}\rangle = -\left(\frac{1}{2\pi}\right)^{m-3}\left(\prod_{i=k+1}^{k+m-3} b_i\right)\times \sin\theta_{k+1}\sin\theta_{k+m-2}\sum_{s_{k+2}=\pm 1}\cdots\sum_{s_{k+m-2}=\pm 1}\left[\prod_{i=k+2}^{k+m-3} s_i(1+s_i\cos\theta_i)\right] I_m^{s_{k+2},\ldots s_{k+m-3}} \quad \text{(S6)}$$

$$I_m^{s_{k+2},\ldots s_{k+m-3}} = \int_{-\pi}^{\pi}\cdots\int_{-\pi}^{\pi}\cos(\lambda_{k+1}+\phi_{k+1,k}-\Psi_{k,k+1})\times \prod_{i=k+2}^{k+m-3}\cos\left[(\lambda_{i-1}+\phi_{i-1,i}-\Psi_{i,i-1})+s_i(\lambda_i+\phi_{i,i-1}-\Psi_{i-1,i})\right]\times \cos(\lambda_{k+m-3}+\phi_{k+m-3,k+m-2}-\Psi_{k+m-2,k+m-3})d\lambda_{k+1}\ldots d\lambda_{k+m-3} \quad \text{(S7)}$$

In eq S6, for uniform notation, we used the same symbols for all coefficients, independent on whether they belong to the fs or to the gs. This modification can be done without the loss of generality or accuracy. The next equations are numbered from S8C on to keep the equation numbers of the original Supporting Information and replace the equations of that Supporting Information from S8 on. Note that, because we consider only a given

pair of atoms of residue i interacting with those at residue $i-1$ or $i+1$, respectively, we now drop atom indices m_i. In what follows $\phi_{i,i-1}$ denotes the base rotation angle of a given atom of residue i interacting with an atom of residue $i-1$ about the $C_i^\alpha \cdots C_{i+1}^\alpha$ virtual-bond axis in the reference system defined by C_{i-1}^α, C_i^α, and C_{i+1}^α and $\phi_{i,i+1}$ is the base rotation angle of a given atom of residue i interacting with an atom of residue $i+1$ about the $C_i^\alpha \cdots C_{i+1}^\alpha$ virtual-bond axis in the reference system defined by C_i^α, C_{i+1}^α, and C_{i+2}^α. Atom indices will be introduced at the end of this section.

By using the Euler formula, eq S7 becomes eq S8C.

$$
\begin{aligned}
I_m^{s_{k+2},\dots s_{k+m-3}} &= \frac{1}{2^{m-2}} \int_{-\pi}^{\pi} \cdots \int_{-\pi}^{\pi} \{\exp[i(\lambda_{k+1}+\phi_{k+1,k}-\Psi_{k,k+1})] + \exp[-i(\lambda_{k+1}+\phi_{k+1,k}-\Psi_{k,k+1})]\} \times \\
&\prod_{j=k+2}^{k+m-3} \Big\{ \exp\left[i(\lambda_{j-1}+\phi_{j-1,j}-\Psi_{j,j-1}) + s_j(\lambda_j+\phi_{j,j-1}-\Psi_{j-1,j})\right] \\
&+ \exp\left[-i(\lambda_{j-1}+\phi_{j-1,j}-\Psi_{j,j-1}) + s_j(\lambda_j+\phi_{j,j-1}-\Psi_{j-1,j})\right] \Big\} \times \\
&\Big\{ \exp\left[i(\lambda_{k+m-3}+\phi_{k+m-3,k+m-2}-\Psi_{k+m-2,k+m-3})\right] \\
&+ \exp\left[-i(\lambda_{k+m-3}+\phi_{k+m-3,k+m-2}-\Psi_{k+m-2,k+m-3})\right] \Big\} d\lambda_{k+1}\dots d\lambda_{k+m-3} = \\
&\frac{1}{2^{m-2}} \int_{-\pi}^{\pi} \cdots \int_{-\pi}^{\pi} \sum_{t_{k+1}=\pm 1} \sum_{t_{k+2}=\pm 1} \cdots \sum_{t_{k+m-3}=\pm 1} \exp\Big[i \sum_{j=k+1}^{k+m-3} (t_j s_j + t_{j+1})\lambda_j \\
&+ t_j s_j(\phi_{j,j-1}-\Psi_{j-1,j}) + t_{j+1}(\phi_{j,j+1}-\Psi_{j+1,j}) \Big] d\lambda_{k+1}\dots d\lambda_{k+m-3}
\end{aligned}
\tag{S8C}
$$

where, for uniform notation, we set $s_{k+1} = 1$. Only those terms in eq S8C give non-zero integrals, for which the coefficients at all λs become zero. This condition results in the following relationships between the ts:

$$\begin{array}{cccc} t_{k+1} & + & t_{k+2} & = 0 \\ s_{k+2}t_{k+2} & + & t_{k+3} & = 0 \\ \vdots & \vdots & \vdots & \vdots \\ s_{k+m-4}t_{k+m-4} & + & t_{k+m-3} & = 0 \end{array} \tag{S9C}$$

and thus

$$t_{k+i} = t_{k+1} \prod_{j=k+2}^{k+i} (-s_j),\ i = 2, 3, \ldots, m-3 \tag{S10C}$$

Setting $t_{k+1} = 1$ or $t_{k+1} = -1$, we obtain the only two sets of ts, with which the contributions to $I_m^{s_{k+2},\ldots s_{k+m-3}}$ are not zero. Clearly, after integration, the two resulting imaginary exponentials will merge into a single cosine term, as given by eq S11C.

$$I_m^{s_{k+2},\ldots s_{k+m-3}} = \frac{(2\pi)^{m-3}}{2^{m-3}} \cos\Bigg\{ -(\Psi_{k,k+1} - \phi_{k+1,k}) + (\Psi_{k+2,k+1} - \phi_{k+1,k+2}) \\ + \sum_{i=k+2}^{k+m-3} \prod_{j=k+2}^{i} (-s_j) \left[-(\Psi_{i,i+1} - \phi_{i+1,i}) + (\Psi_{i+2,i+1} - \phi_{i+1,i+2}) \right] \Bigg\} \tag{S11C}$$

By plugging in the relationship

$$\gamma_i = \Psi_{i+1,i} - \Psi_{i-1,i} + \pi \tag{S12C}$$

the integrals of eq S7 are expressed by eq S13C.

$$I_m^{s_{k+2},\ldots s_{k+m-3}} = (-\pi)^{m-3} \cos\left[(\gamma_{k+1} + \delta_{k+1}) + \sum_{i=k+2}^{k+m-3} \prod_{j=k+2}^{i} (-s_j)(\gamma_i + \delta_i) \right] \tag{S13C}$$

$$\delta_i = \phi_{i,i-1} - \phi_{i,i+1} \tag{S14C}$$

Plugging eq S13C into eq S6 and then eq S6 into eq S7 and, finally eq S7 into eq S1

and applying recursively the reduction formulas for cosines of different angles to get the same phase angles in all terms, canceling the $(-1)^{m-3}$ factors, and defining

$$C_i = \sqrt{\left(\sum_{m_i} b_{i,m_i} c_{i,m_i} \cos\delta_{i,m_i}\right)^2 + \left(\sum_{m_i} b_{i,m_i} c_{i,m_i} \sin\delta_{i,m_i}\right)^2} \quad \text{(S15C)}$$

$$\cos\Phi_i = \frac{1}{C_i}\sum_{m_i} b_{i,m_i} c_{i,m_i} \cos\delta_{i,m_i} \quad \text{(S16C)}$$

$$\sin\Phi_i = \frac{1}{C_i}\sum_{m_i} b_{i,m_i} c_{i,m_i} \sin\delta_{i,m_i} \quad \text{(S17C)}$$

we finally obtain eq 3C of the main text of this Correction.

Derivation of equation 6C

Eq 6C can be written as eq S18C.

$$U_m = \left(\frac{1}{2}\right)^{m-3} \prod_{i=k+2}^{k+m-3} C_i \sin\theta_{k+1} \sin\theta_{k+m-2} W_{mk} \quad \text{(S18C)}$$

where

$$W_{mk} = \sum_{s_{k+2}=\pm 1} \sum_{s_{k+3}=\pm 1} \cdots \sum_{s_{k+m-3}=\pm 1} \left(\prod_{i=k+2}^{k+m-3} s_i\right) (1 + s_i \cos\theta_i)$$
$$\cos\left[(\gamma_{k+1} + \Phi_{k+1}) + \sum_{i=k+2}^{k+m-3} \prod_{j=k+2}^{i} (-s_j)(\gamma_i + \Phi_i)\right] \quad \text{(S19C)}$$

For ideal folded chain segments, $\theta_i = 90°$, $i = k+2, k+3, \ldots k+m-3$ and, thus $(1 + s_i \cos\theta_i) = 1$, $i = k+2, k+3, \ldots k+m-3$. Setting the index of the first residue in the segment at 1 (which can be done without the loss of generality), we obtain eq S20C.

$$W_m = \sum_{s_3=\pm1}\sum_{s_4=\pm1}\cdots\sum_{s_{m-2}=\pm1}\prod_{i=3}^{m-2} s_i \cos\left[(\gamma_2+\Phi_2)+\sum_{i=3}^{m-2}\prod_{j=3}^{i}(-s_j)(\gamma_i+\Phi_i)\right] \tag{S20C}$$

By expanding the summation over s_{m-2}, we obtain eq S21C and making use of the identity $\cos(x \pm y) = \cos x \cos y \mp \sin x \sin y$, we obtain eq S23C.

$$\begin{aligned} W_m = &\sum_{s_3=\pm1}\sum_{s_4=\pm1}\cdots\sum_{s_{m-2}=\pm1}\prod_{i=3}^{m-3} s_i \times \\ &\left\{\cos\left[(\gamma_2+\Phi_2)+\sum_{i=3}^{m-3}\prod_{j=3}^{i}(-s_j)(\gamma_i+\Phi_i)-\prod_{j=3}^{m-3}(-s_j)(\gamma_{m-2}+\Phi_{m-2})\right]\right. \\ &\left.-\cos\left[(\gamma_2+\Phi_2)+\sum_{i=3}^{m-3}\prod_{j=3}^{i}(-s_j)(\gamma_i+\Phi_i)+\prod_{j=3}^{m-3}(-s_j)(\gamma_{m-2}+\Phi_{m-2})\right]\right\} \end{aligned} \tag{S21C}$$

$$\begin{aligned} = 2&\sum_{s_3=\pm1}\sum_{s_4=\pm1}\cdots\sum_{s_{m-3}=\pm1}\prod_{i=3}^{m-3} s_i \sin\left[(\gamma_2+\Phi_2)+\sum_{i=3}^{m-3}\prod_{j=3}^{i}(-s_j)(\gamma_i+\Phi_i)\right]\times \\ &\sin\left[\prod_{j=3}^{m-3}(-s_j)(\gamma_{m-2}+\Phi_{m-2})\right] \end{aligned} \tag{S22C}$$

$$= 2(-1)^{n-3}\sum_{s_3=\pm1}\sum_{s_4=\pm1}\cdots\sum_{s_{m-2}=\pm1}\sin\left[(\gamma_2+\Phi_2)+\sum_{i=3}^{m-3}\prod_{j=3}^{i}(-s_j)(\gamma_i+\Phi_i)\right]\sin(\gamma_{m-2}+\Phi_{m-2}) \tag{S23C}$$

When proceeding from eq S22C to eq S23C, we took used the following two identities:

$$\sin\left[\prod_{j=3}^{m-3}(-s_j)(\gamma_{m-2}+\Phi_{m-2})\right] = \prod_{j=3}^{m-3}(-s_j)\sin(\gamma_{m-2}+\Phi_{m-2}) \tag{S24C}$$

$$\prod_{j=3}^{m-3} s_j \prod_{j=3}^{m-3}(-s_j) = (-1)^{m-5} = (-1)^{m-3} \tag{S25C}$$

Subsequent summation over $s_{m-3}, s_{m-4}, \ldots, s_3$ and utilizing the identity $\sin(x+y)+$

$\sin(x-y) = 2\sin x \cos y$ yields eq S26C.

$$W_m = (-1)^{m-3} 2^{m-3} \sin(\gamma_2 + \Phi_2) \prod_{i=3}^{m-3} \cos(\gamma_i + \Phi_i) \sin(\gamma_{m-2} + \Phi_{m-2}) \qquad \text{(S26C)}$$

which, after restoring the index of the first and the last residue and plugging the resulting W_{mk} into eq S18C yields eq 6C of the main text of this Correction.

References

(1) Sikorska, C.; Liwo, A. Origin of Correlations between Local Conformational States of Consecutive Amino Acid Residues and Their Role in Shaping Protein Structures and in Allostery. *J. Phys. Chem. B* **2022**, *126*, 9493–9505.
